# Elucidating the Role of Electric Fields in Fe Oxidation via an Environmental Atom Probe

**DOI:** 10.1002/anie.202423434

**Published:** 2025-03-21

**Authors:** Sten V. Lambeets, Naseeha Cardwell, Isaac Onyango, Mark G. Wirth, Eric Vo, Yong Wang, Pierre Gaspard, Cornelius F. Ivory, Daniel E Perea, Thierry Visart de Bocarmé, Jean‐Sabin McEwen

**Affiliations:** ^1^ Physical and Computational Sciences Directorate Pacific Northwest National Laboratory 902 Battelle Blvd 99354 Richland WA United States of America; ^2^ The Gene and Linda Voiland school of Chemical Engineering and Bioengineering Washington State University 1505 Stadium Way 99164 Pullman WA United States of America; ^3^ Environmental Molecular Sciences Laboratory Pacific Northwest National Laboratory 902 Battelle Blvd 99354 Richland WA United States of America; ^4^ Institute for Integrated Catalysis Pacific Northwest National Laboratory 902 Battelle Blvd 99354 Richland WA United States of America; ^5^ Centre for Nonlinear Phenomena and Complex Systems Université Libre de Bruxelles Campus Plaine CP231 B-1050 Brussels Belgium; ^6^ Chemistry of Surfaces, Interfaces and Nanomaterials Université Libre de Bruxelles Campus Plaine CP243 B-1050 Brussels Belgium; ^7^ Departments of Physics and Astronomy/Chemistry/Biological Systems Engineering Washington State University 99164 Pullman WA United States of America

**Keywords:** Atom Probe Tomography, Field Ion Microscopy, First Principles Calculations, Oxidation, Iron, Operando

## Abstract

We quantify the effects of intensely applied electric fields on the Fe oxidation mechanism. The specimen are pristine Fe single crystals exposing a variety of surface structures identified by field ion microscopy. These crystals are simultaneously exposed to low pressures of pure oxygen gas, on the order of 10^−7^ mbar, while applying intense electric fields on their surface of several tens of volts per nanometer. The local composition of the different surface structures is probed directly and in real time using an Environmental Atom Probe and successfully compared with first principles‐based models. We found that rough Fe{244} and Fe{112} facets are more reactive toward oxygen than compact Fe{024} and Fe{011} facets. Results demonstrate that the influence of an electric field on the oxidation kinetics depends on the timescales that are involved as the system evolves toward equilibrium. The initial oxidation kinetics show that strong increases in electric fields facilitate the formation of an oxide. However, as one approaches equilibrium, high field values mitigate this formation. Ultimately, this study elucidates how high externally applied electric fields can be used to dynamically exploit reaction dynamics at the nanoscale towards desired products in a catalytic reaction at mild reaction conditions.

## Graphical abstract

Field‐Assisted Fe Oxidation imaged by Environmental Atom Probe. The oxidation of iron (Fe) at the tip of a sharp needle under high electrostatic fields has been visualized using an Environmental Atom Probe (EAP) and corroborated through Density Functional Theory (DFT) calculations and a mean‐field model applied to a multi‐faceted catalytic grain, alongside statistical‐mechanical analyses. EAP observations underscore the impact of surface structures, revealing that Fe oxidation predominantly occurs on exposed facets such as Fe{244} and Fe{112}, while facets with higher coordination numbers like Fe{024}, Fe{013}, and Fe{011} exhibit comparatively lower levels of oxidation. Intense electrostatic fields exert a significant influence on Fe oxidation by polarizing and attracting gas molecules toward the surface, thereby effectively raising the local pressure via enhanced electric field concentrations at the tip‘s apex.

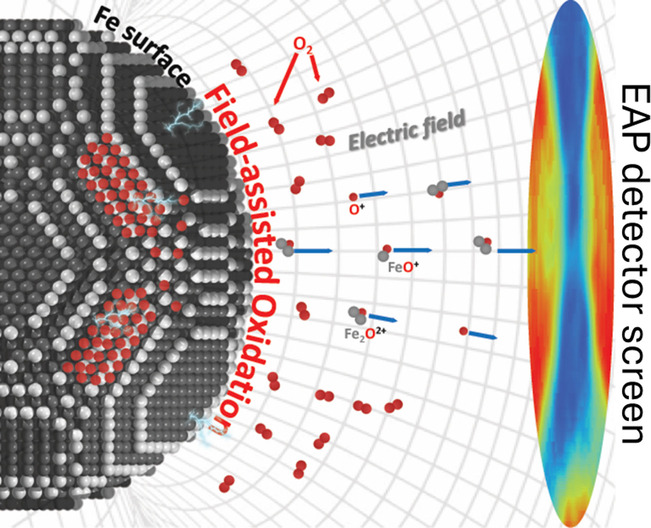

## Introduction

Heterogeneous catalysis remains a complex and poorly understood process. Researchers often design catalysts with limited fundamental knowledge of their underlying mechanisms. Advancements in real‐time observation of surface reactions could significantly enhance our understanding, leading to more systematic and informed catalyst design.

Amongst other surface science techniques, the atom probe microscopy family of techniques are capable of near‐atomic scale imaging of surfaces, mimicking single hemispherical nanoparticle materials relevant to heterogeneous catalysis. Field emission microscopy (FEM) has been used to observe nanoscale surface reactions in real‐time.[^1^, ^2^, ^3^, ^4^, ^5^, ^6^, ^7^, ^8^, ^9^, ^10^, ^11^, ^12^, ^13^] Similarly, surface reaction studies have been performed with field ion microscopy (FIM) at high temperature (450 K).^[8]^ In both cases, FEM and FIM do not allow for an easy chemical characterization of the observable. Efforts have been conducted to try to correlate work function variations observed in FEM with surface species formation.[^14^, ^15^] Although recent approaches have opened chemical identification capabilities with FIM when combined with atom probe capabilities, such approaches are performed at low temperature making it incompatible with the goal of observing real‐time reactions.[^16^, ^17^]

Atom probe tomography (APT) enables the chemical identification of species but is usually performed at cryogenic temperatures and is neither natively built for real‐time observations nor for surface analyses. In this work, we present the environmental atom probe (EAP) approach that consists of using an APT system in the presence of reactive gases at ambient temperature and show that APT can be adapted to monitor the oxidation of Fe in real‐time and with nanometer resolution. Fe is an excellent earth‐abundant material candidate for the hydrodeoxygenation (HDO) of bio‐oil.[^18^, ^19^, ^20^] Monitoring the reaction in real‐time in the presence of an applied electric field (EF) allows for the assessment of how the oxidation of Fe is influenced by its presence and, ultimately, how EFs can be used toward controlled reaction dynamics.[^18^, ^19^, ^20^] Furthermore, understanding how electric fields affect reaction dynamics can also provide fundamental insights into mitigating the oxidation of Fe since the desirable catalytic properties of Fe are often impeded by its oxidation.^[20]^

A significant number of recent investigations revealed the importance of EFs in surface chemistry to the point that it became a key parameter to consider.[^21^, ^22^, ^23^, ^24^, ^25^, ^26^, ^27^] Previous work has shown how EFs can dramatically enhance nickel's coke resistance when used in methane steam reforming at lower temperatures and pressures.^[24]^ Similarly, the application of EF's on single‐atom catalysts (SACs) for the nitrogen (N_2_) reduction reaction (NRR) to ammonia (NH_3_) has shown promising results, opening a new path to develop strategies for low temperature NH_3_ synthesis.^[21]^ Additionally, the remarkable consequences of EFs on surface chemistry has been reported by Kreuzer et al.^[25]^ and Kathmann^[27]^ who demonstrated how EFs can modify molecular orbitals to the extent that it will give a different electronic, and consequently, a different chemical “envelope” to the targeted molecule (e.g., N_2_ chemically behaving like CO). Che et al. demonstrated how the polarity of the EF directly influences the chemical and surface properties of Ni.^[24]^

EFs over 1 V/nm can alter molecular orbital energies of adsorbates, adsorption structures, vibrational frequencies, and oxidation states.[^25^, ^27^, ^28^] They also change activation energies and, hence, the associated kinetic parameters. If the application of EFs undoubtedly plays a central role in surface chemistry, their influence on surface chemistry mechanisms and the EF intensity required to trigger those modifications remains to be unraveled. Atom Probe (AP) techniques typically work with EFs on the order of 1 to 60 V/nm and are thus well suited for such studies.

In this work, we demonstrate the effects of EFs on the O_2_/Fe system using an EAP. All experiments have been executed at 303 K by exposing pure Fe surfaces to O_2_ within a pressure range between 10^−9^ to 10^−7^ mbar. We examine the effects of the intensity of the EF on the evolution of the surface composition, as well as the local distribution of Fe oxidation in real time. Finally, we compare our experimental observations with theoretical models before ending this work on a discussion focused on the future of EAP technical development and its role as a tool toward the investigation of EFs on enhancing chemical reactions.

## Results and Discussion

### Field Ion Microscopy of Fe

Prior to oxidation and subsequent analysis, Fe surfaces in the form of needles are imaged using FIM. In this work, we used digital FIM, also known as “eFIM”,[^29^, ^30^] equipped on modern APT apertures, producing lower quality images than conventional FIM equipped with phosphorescent screens.[^31^, ^32^] Despite the relatively low quality of the FIM image, it is still possible to approximatively identify the visibly different oriented facets by their Miller indices by comparing FIM images with a stereographic projection on a ball model.

**Figure 1:**
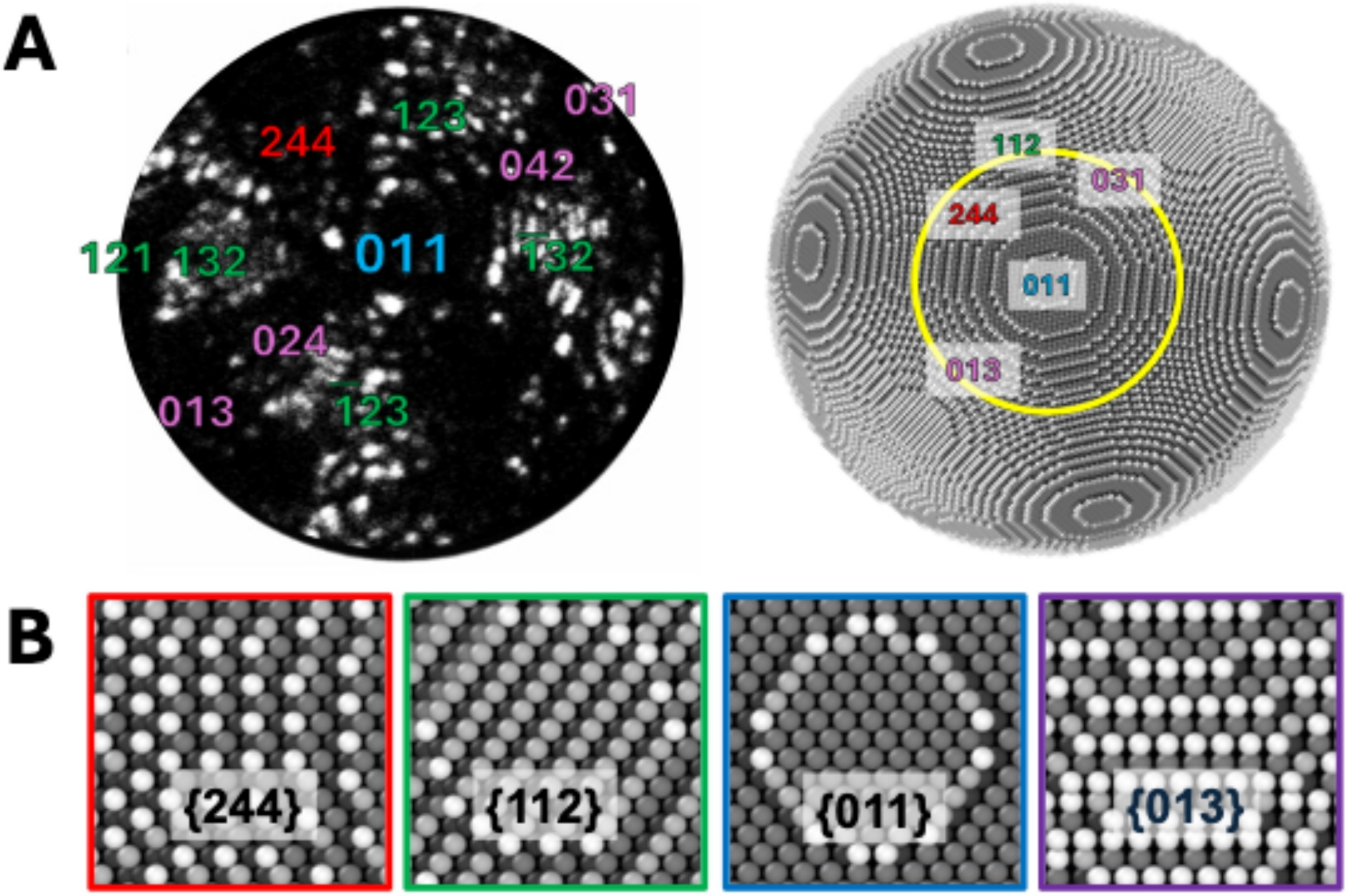
(A) FIM image (left) and associated ball model (right) of a (011)‐oriented Fe needle specimen. The ball model is that of a sphere. Conditions of acquisition: 45.0 K, 3300 V (~35 V/nm), P(Ne)=2.2×10^−6^ mbar. Main facets identified are Fe{112}, Fe{013}, Fe{244} and a central Fe(011). (B) Ball model representations of Fe{011}, {244}, {112} and {013} selected facets taken from the spherical ball model. The shades of grey in the ball model are proportional to the coordination number of the atoms, with light grey spheres representing a coordination number of 4 and dark grey spheres representing a coordination number of 8.

Figure 1 shows a specimen identified as a (011)‐oriented tip. The central (011) pole is surrounded by two pairs of {013}, {024} and {244} facets, and four {112} facets spread in a rectangle around the central (011). Fe{112} facets are distinguishable on the FIM image while the other structures remain widely dark. Note that such a field emitter needle tip morphology approximates a single catalytic grain where a variety of facets are simultaneously exposed and analyzed as can be seen through the comparison to the ball models.

### APT at 303 K

APT is a 3D atomic scale composition mapping technique predicated on field evaporation,^[33]^ the process by which surface atoms are emitted as ions under pulsed applied electric fields. The mass‐to‐charge‐state ratios of field evaporated species are determined from time‐of‐flight (ToF) and their relative position on the specimen surface from where they originated is determined from a position‐sensitive detector. APT combines the position information obtained from the sequence of detected ionic impacts on a position‐sensitive detector, with ToF chemical identification to reconstruct a 3D composition point cloud map of the specimen.[^33^, ^34^] In this work APT is used in the traditional sense to field evaporate Fe surface atoms at a controlled rate to remove surface adsorbates and any damage from the specimen preparation process to reveal a pristine Fe surface for subsequent controlled surface reaction studies observed in real‐time.

We used the detection of field‐evaporated Fe to estimate the applied EF over the Fe surface. At 0 K, Fe evaporation field is ~35 V/nm for an Fe^+^ ion.[^33^, ^34^, ^35^, ^36^] However, it is important to acknowledge the complexity to define a fixed value of the evaporation field of Fe under non‐equilibrium conditions. First‐principles calculations estimate the electric fields required to trigger Fe field evaporation, ranging from 33 to 44 V/nm. The lower field value corresponds to field evaporation from a Fe(001) facet, while the higher value is observed at a step site.[^36^, ^37^, ^38^] In addition to the dependence on the surface structure,[^39^, ^40^] field evaporation is a thermally activated process, requiring lower applied EFs at higher temperatures, meaning less applied voltage is necessary to extract Fe ions at elevated temperatures.[^41^, ^42^] With the approach described in Section 1.1.3 of the SI, we estimated an average electric field of ~24.8 V/nm.

### Field‐Assisted Fe Oxidation Model

#### Field‐Dependent Adsorption Energies

**Figure 2:**
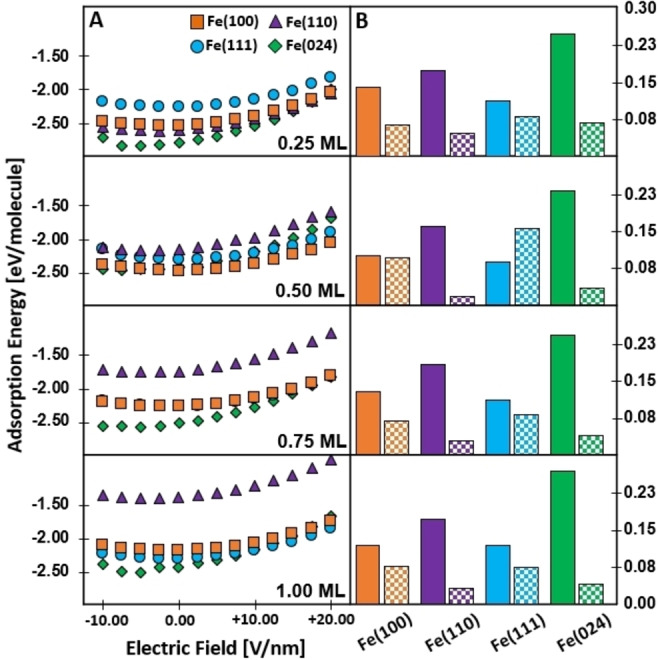
(A) Calculated E_ads_ of oxygen atoms on iron at the different coverages for the four facets. The electric field was varied from −10.0 V/nm to +20.0 V/nm in increments of 2.5 V/nm. (B) Changes in E_ads_ by application of a positive 10.0 V/nm (solid bar) or negative 10.0 V/nm (checkered bar) electric field. Changes are in reference to no electric field applied. All coverages tested.

To correlate the effects of an EF with EAP experimental observations, we performed density functional theory (DFT) calculations of the adsorption energies (E_ads_) of oxygen atoms on four chosen facets. We assume that there are no activation barriers separating the precursor and the chemisorbed state since such a model is consistent with the experimental results that are available in the literature (see Supporting Information Section 2.5.2). Four levels of coverage were studied as a function of applied EF between ‐10.0 V/nm and +20.0 V/nm with 2.5 V/nm increments (Figure 2A); calculation details are found in Supporting Information Section 2.1. Note that these are 0 K ground state DFT calculations for which the geometry has been optimized, for which the DFT‐based parameters will be incorporated in a simple microkinetic model (see Eqn. S41) where temperature, pressure, and electric field effects will be assessed. These calculations provide quantitative insight into the impact of electric fields on O adsorption and highlight the differing behaviors between the facets which comprise the whole Fe grain. Moreover, they are essential in defining a function for the coverage‐ and facet‐dependent O adsorption energy, which are then used, along with Kubic Harmonics,^[43]^ to construct kinetic equations that lead to the development of the multifaceted grain model.

We observe: (1) the E_ads_ varies along a parabolic function of the applied EF, consistent with literature findings,^[20]^ where the largest Root Mean Squared Error (RMSE) is 24 meV/molecule, establishing that our model accurately captures the oxygen E_ads_ dependence on EFs regardless of coverage or facet‐type; (2) except for the {024} facet, the most negative E_ads_ is found at 0 V/nm, indicating that EFs are vital for mitigating oxygen adsorption on Fe and therefore Fe oxidation; (3) the EF affects compact facets like Fe{011} greater than more open facets like Fe{111}.

To further investigate the impacts of EFs on oxygen adsorption to the Fe surface, a comparison of the E_ads_ differences from no applied EF (+0.0 V/nm) to the application of a positive applied EF (+10.0 V/nm), shown in solid‐colored bars, and the application of a negative EF (−10.0 V/nm), shown in checkered bars, was examined for all coverage‐facet combinations (Figure 2B). We find that the change in E_ads_ is largest for the positively applied field compared to the negatively applied field for all coverage‐facet combinations (i.e. the solid bars are larger than the checkered bars across the full coverage and facet range). This suggests that applying a positive electric field (EF) has a more significant impact on reducing oxygen adsorption than applying a negative field, regardless of the oxygen coverage value. Fe(111) shows the smallest change when a positive EF is applied (about 0.11 eV, compared to 0.12 eV for Fe(100), 0.17 eV for Fe(110), and 0.25 eV for Fe(024)). In contrast, it exhibits the largest change with a negative EF (around 0.10 eV, compared to 0.08 eV for Fe(100), 0.03 eV for Fe(110), and 0.05 eV for Fe(024)). This indicates that positive and negative EFs have distinct effects on lateral interactions in these O/Fe systems. Additionally, we note that the application of a negative field of this amplitude on the Fe specimen would provoke additional effects, such as the field emission of electrons from the surface.^[44]^

#### Spatial‐Time Conversion and Constant Electric Field

EAP consists of performing an APT analysis at constant voltage while exposing the specimen to reactive gases—oxygen in the current studies. In EAP, both a constant DC electric field is applied near but below the field evaporation threshold of the Fe substrate while still strong enough to induce oxygen adsorption, and a pulsed electric field is periodically applied to provoke the field evaporation of the oxide products. The complete experimental workflow for EAP can be found in Section 3.2 of the SI. In practice, achieving a perfect threshold across the entire surface of the apex is virtually impossible. The different surface structures exposed along the apex generate varying local electric fields, which lead to differential field evaporation across the surface. Additionally, the introduction of gases such as O_2_ ^[45]^ lowers the evaporation field of the substrate. This occurs because O_2_ interacts with the Fe surface, aiding in the removal of Fe surface atoms. As a result, the interaction between Fe and O_2_ is observed in the form of Fe_2_O^n+^ ions. Note that we refer to the “constant EF” as the applied electric field maintained between two electric pulses, and the “pulsed EF” as the intensity of the applied electric field when the electric pulse is applied. More details on the EAP principles and operation can be found in the Supporting Information Section 1.1. An EAP result is thus a 3D atom map but in an “(x,z,t)” space instead of an “(x,y,z)” space for APT. An EAP atom map can be seen as the composition of the specimen surface over time. The utilization of this approach requires the assumptions that: (1) the quantity of surface atoms removed does not heavily impact the global geometry of the substrate such that it conserves its radius curvature and its crystallography; (2) the global applied EF remains unchanged if the applied voltage is constant.

**Figure 3:**
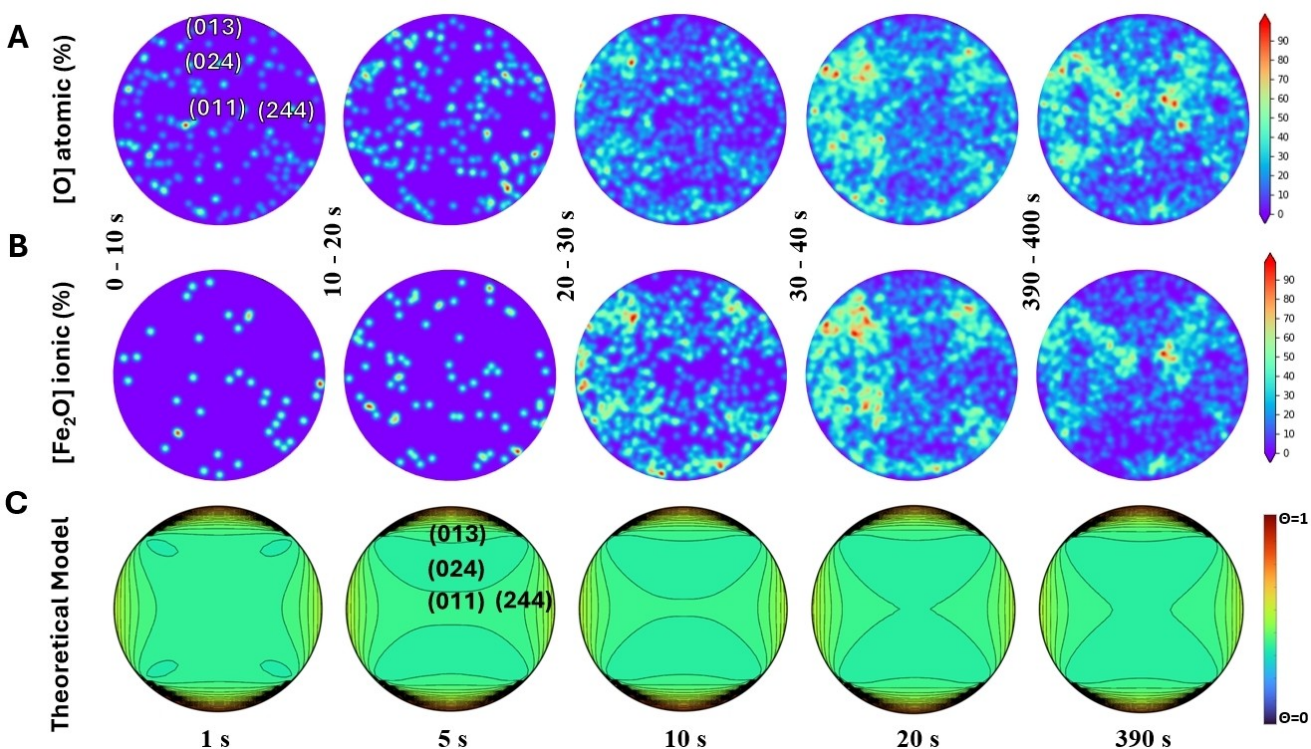
Experimentally measured 2D projection composition maps of (A) O(%)atomic, (B) Fe_2_O^n+^ ionic of a Fe specimen exposed to 1.2×10^−7^ mbar of pure O_2_ with a constant ~18 V/nm (~23.4 V/nm pulsed) applied EF estimation at 303 K. The experimental composition maps shown are displaying the average composition detected over a 5 min time‐lapse (C) Theoretically derived 2D projection composition maps from corresponding first principles‐based model of evolving oxygen coverage over a hemispherical field emitter tip with an EF of 24.12 V/nm, a pressure of 1.1×10^−8^ mbar and a temperature of 300 K. We note that the displayed contour lines were generated automatically within the plotting software. Corresponding images at fixed contour line values are shown in Figure S17.

The oxygen concentration, reported in Figure 3A as the proportion of O atoms detected vs the total amount of collected atoms, indicates the heterogeneous formation of Fe oxides over specific faceted regions of the Fe surface. O atoms are mainly detected in the form of Fe_2_O^n+^ and FeO^n+^ ions (with *n*=1,2) found at 64 Da and 72 and 36 Da (Mass spectra found in Figure S25). Additionally we find other sources of O atoms in O^+^ detected at 16 Da whose detection may be the result of straight field evaporation of surface activated O or from post field dissociation of O_2_ into atomic oxygen species. With increasing time after 30 min of exposure, more O atoms are found over open facets like Fe{112} and Fe{244} than on compact facets like Fe{013} and Fe{011}, which is consistent with the previous observation of G. Cranstoun and J.T. Lynch with FIM.^[4]^ Ionic concentration plots of Fe_2_O^n+^ (Figure 3B), Fe^n+^ and FeO^n+^ clearly display the complementary distribution between those three species (Figure S3). Starting from a pure Fe surface, Fe^n+^ are mainly detected over the Fe{013} regions while Fe_2_O^n+^ are distributed over Fe{244} and {112}, clearly showing experimentally which facets are more prone to initiate oxide formation.

To compare the experimental results with our first principles‐based model, Kubic Harmonics^[43]^ were used to expand the E_ads_ of oxygen on a multifaceted Fe surface. The tip was modeled using a hemispherical geometry, where the EF strength along the hemisphere has been shown to be a good estimation of field distribution on the tip in experiment.^[46]^ However, we do note that the spherical model is not the final, equilibrium geometry of the tip, but remains a good estimation for the tip geometry for the field of view. The details for the creation of these plots can be found in the SI, Section 2.2. The normalized surface oxygen distribution on the Fe crystal was simulated over a period of up to 18,000 seconds, with the first 400 seconds shown in Figure 3, at an oxygen partial pressure of 1.1×10^−8^ mbar.^[43]^ Plots constructed using adsorption energies presented in Figure 2 qualitatively agree with experiment but the electric field and temperature (30.5 V/nm and 700 K^[43]^) for the simulations (Figure S18) substantially differ from experiment (~23.4 V/nm and 303 K). O adsorption on the four facets in Figure 2 did not take into account the formation of a thin oxide layer, which we found to already be present at the start of the experiment, and so the overall oxygen adsorption strength is likely overestimated. DFT calculations on the adsorption of atomic O on an Fe oxide, FeO(001), found the adsorption energy on an Fe surface oxide to significantly decrease with increasing oxygen coverage (Figure S8) in comparison to a non‐oxide Fe surface (by 1.49 eV, 1.13 eV, 0.54 eV, and 0.38 eV for the 0.25 ML, 0.50 ML, 0.75 ML, and 1.00 ML cases, respectively). Even as we apply EFs, the adsorption energy is much smaller (with an average decrease in adsorption energy of 1.4 eV across the full, tested EF range at 0.25 ML) (Figure S9). Applying antiferromagnetic (AFM) ordering on the FeO surface oxide reduces the oxygen adsorption energy even further (Figure S10). When the adsorption energy was uniformly reduced by 1.2 eV across all crystal facets during the construction of the multifaceted plots, the parameters (24.12 V/nm and 303 K) aligned well with those used in the experiment (Figure 3). The corresponding local oxygen coverage over the Fe surface is displayed at different timeframes in Figure 3C and compared to the experimental findings in Figure 3A. As can be seen from the plots, there is a depletion of the oxygen coverage around the {024} facets while there is a higher oxygen coverage as one approaches the {013} facets as one approaches the flanks of the tip. The decrease of the oxygen coverage around the {024} correlates well with the fact that the oxygen binding energy decreases significantly on this facet in the presence of a positive field. Further, as discussed in detail in the SI, EFs can have the effect of increasing the pressure at the apex of the specimen tip as compared to the flanks.[^26^, ^47^] Videos of the oxygen distribution are included in the SI. Experimentally, we find that the formation of an oxide occurs over Fe{244} while the Fe{013} facets remain unscathed by the oxidation.

As such, atom probe systems offer the possibility to study chemical phenomena under intense EFs. Applied EFs have multiple impacts on chemical systems that can either promote or inhibit a given reaction pathway.

#### Determining the Effects of Electric Fields on Fe Oxidation

**Figure 4:**
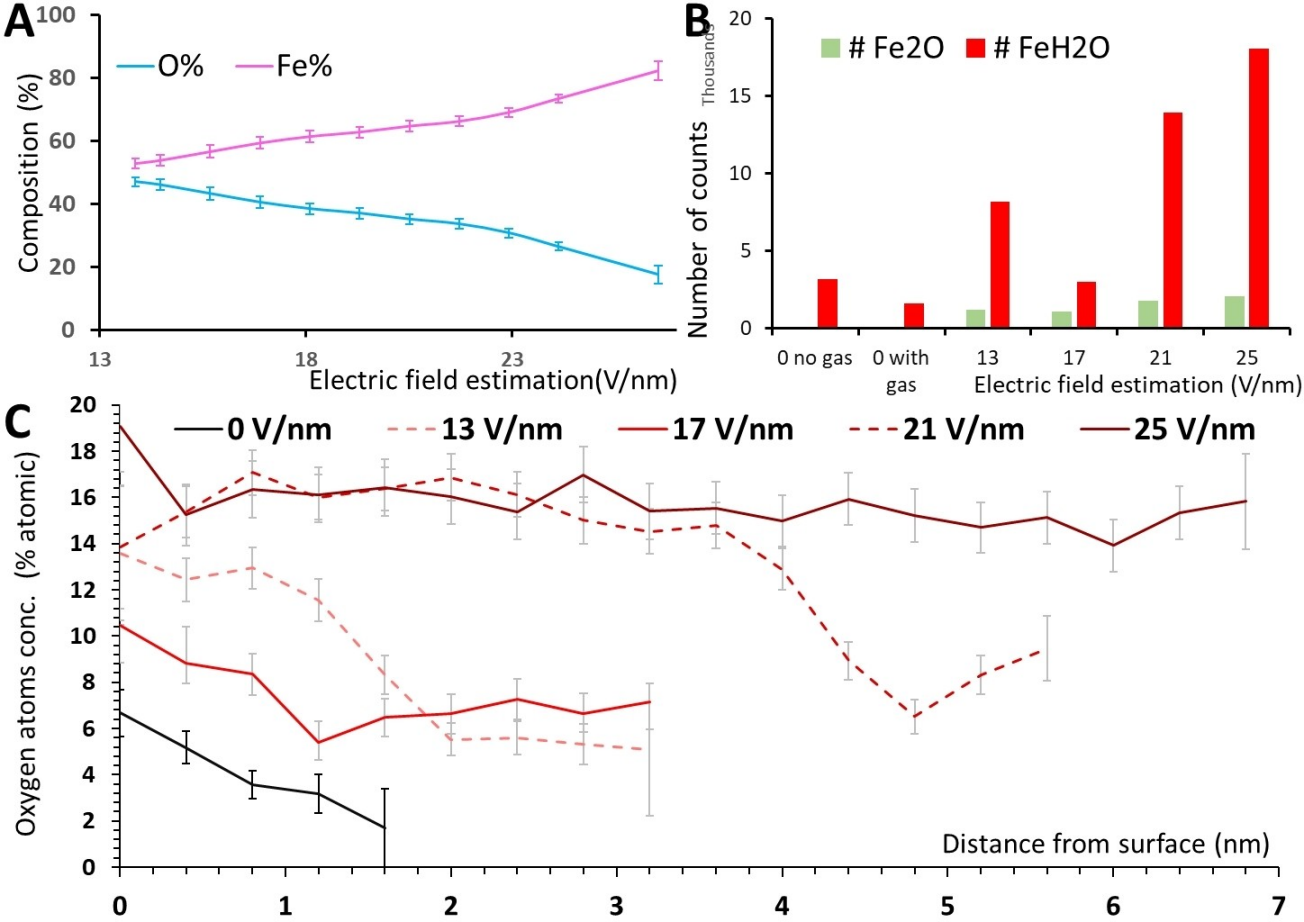
(A) EAP measurement of the Fe surface composition while the specimen is exposed to 1.2×10^−9^ mbar of pure O_2_ and with different applied EFs at 303 K. The O at.% and Fe at.% are obtained by decomposing the detected ions (Fe^n+^, O^n+^, Fe_2_O^n+^ and FeO^n+^) to individual atoms. (B) Average O atoms composition (%) of the entire APT analysis after 2.5×10^−9^ mbar of O_2_ exposure at 303 K for 30 min with varying applied EFs on the Fe surface. (C) 1D O at.% composition profile within the first atomic layers of Fe(121) after the same oxygen exposure described in B. The region of interest is a cylinder of 10 nm dia. going across the O‐enriched region located over the Fe(121) facet. The 1D concentration profile is calculated along the cylinder with a bin size of 0.4 nm. An example is illustrated in Figure S4 of the SI.

To elucidate the dynamic effects of EFs on Fe oxidation, two sets of experiments were conducted. In the first set of experiments, the Fe specimen has been exposed to a constant oxygen pressure of 1.2×10^−9^ mbar while the magnitude of the constant applied EF was progressively varied from 13 to 25 V/nm and then back down from 25 to 13 V/nm. The average atomic surface composition found during these EAP experiments is reported in Figure 4A. We find a direct correlation between the apparent surface composition of oxygen and the intensity of the EF. A higher evaporation field promotes the removal of surface oxygen atoms and thus prevents the accumulation of O(ads) species.

The second set of experiments consist of exposing the Fe specimen to 2.5×10^−9^ mbar of O_2_ while applying an EF of varying magnitude for 30 min followed by a regular APT analysis at 303 K to quantify the amount of O accumulated within the specimen during the exposure. The applied potentials correspond to a constant applied EF of 13, 17, 21 and 25 V/nm. Oxygen atoms are detected whether an EF is applied or not, particularly under the form of water contamination, but their relative quantity increases with EF intensity (Figure 4B). However, these plots fail to show the spatial heterogeneity of oxide formation. Oxygen atoms are mainly detected in the form of FeH_2_O^2+^ (37 Da) and Fe_2_O^2+^ (64 Da) ions along specific regions of the three dimensional reconstruction identified as covering the Fe{112} facets (Figure S4). 1D composition profiles of O atoms along the Fe(121) facet, after exposure using the EFs of varying magnitude, are depicted in Figure 4C and show atomic compositions reaching up to 30 % oxygen near the surface after oxygen exposure with 25 V/nm.

Our observations suggest that EFs have a promotor effect on the oxidation process. In the presence of high EFs of several tens of V/nm, nearby gas molecules, such as O_2_, become polarized[^48^, ^49^]—the same effect is used in FIM.^[47]^ Such an effect is also relevant to explain the attraction of remaining water molecules in the chamber. With increasing intensity of the applied EFs, the velocity at which the gas molecules interact with the surface increases, thus increasing the probability of reaction between the O_2_(g) and the Fe surface. This effect can be seen as an effective increase of the gas pressure on the surface.^[26]^ Molecular polarization of O_2_(g) is thus identified as a positive effect of the EF on Fe oxidation. Such oxidation enhancement in the presence of an EF is consistent with previous investigations on a Rh field emitter tip, where the effect of an external field on oxygen incorporation into the surface of an oxide layer was quantified,[^8^, ^10^] and with the field‐assisted W oxidation conducted by Nowak et al. with TEM and APT.^[50]^

#### Normalized Oxygen Distribution on the Fe Surface

**Figure 5:**
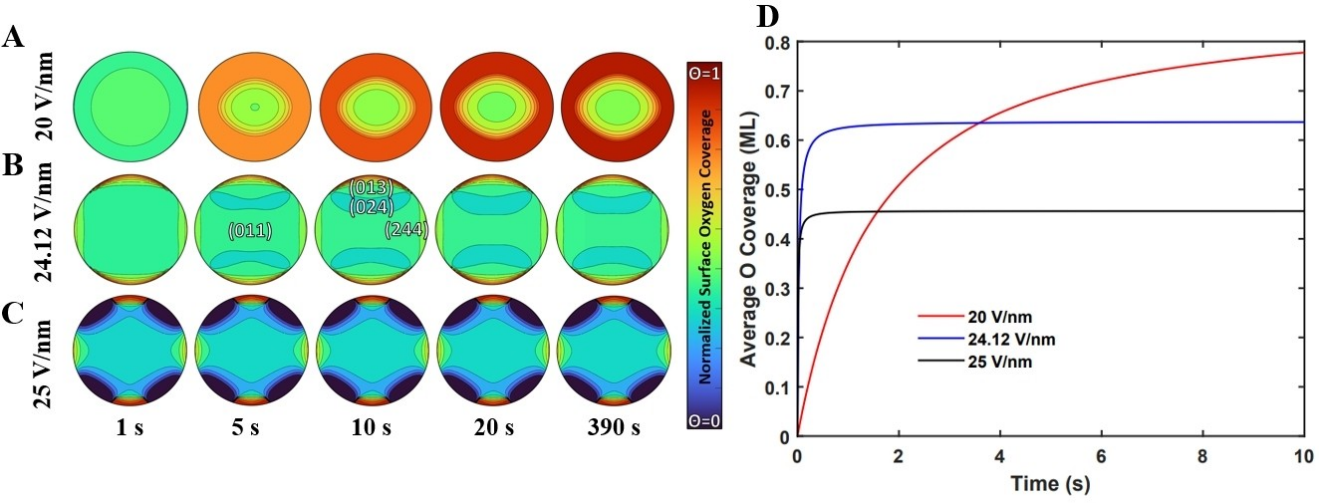
Snapshots of the time evolution of oxygen coverage on the Fe crystallite at 1.1×10^−8^ mbar and 300 K under (A) 20 V/nm, (B) 24.12 V/nm and (C) 25 V/nm applied electric fields. (D) Time evolution of oxygen coverage under varying electric fields where the average coverage of the entire hemispherical field emitter tip is taken into account. We note that the displayed contour lines were generated automatically within the plotting software.

The oxygen distribution was simulated at 20, 24.12 and 25 V/nm (Figure 5A–D)—these values were chosen as they fall near the experimentally tested range of pulsed EFs. Focusing on the 20 V/nm case, starting with an initially clean Fe tip surface, indicated by the deep blue color (i.e., oxygen‐free surface), the surface becomes significantly covered with oxygen after 20 s, with a lower concentration over the central Fe(011) facet compared to the peripheral structures. Progressively, the oxygen coverage pattern intensifies after 20 s and maintains this general shape throught the duration of the simulation.

We note that the coverage around the Fe{024} facets is less as compared to the Fe{244} facets as is seen with the EAP observations. Further, low‐coordination open facets such as Fe{244} are particularly responsive to oxygen adsorption as compared to high‐coordination Fe{024} facets. Hence, larger oxygen coverage is expected over Fe{244} as compared to Fe{024}. At 20 V/nm and 24.12 V/nm, such a decrease in the oxygen coverage on the Fe{011} is not observed in our multi‐faceted Fe grain model. This is likely due to the higher oxygen partial pressure at the apex of the tip than at its periphery.

This effect is less pronounced with an EF of 24.12 V/nm (Figure 5B). Besides common general trends with increasing oxygen coverage and facet‐dependent distribution of this coverage, we find important differences at this higher EF if we take into account all of the facets that are simultaneously exposed on the field emitter tip (not just those shown in Figure 5A and Figure 5B). Firstly, the initial coverage distribution (after 1 s) is higher at 24.12 V/nm as compared to the 20.0 V/nm case (Figure 5D), indicating that by only increasing the EF, the initial oxygen adsorption is drastically increased.

The equilibrated oxygen coverage pattern also appears earlier at a field value of 24.12 V/m as compared to when a field of 20 V/m is applied and corresponds to the EAP observations with a high oxygen coverage on the Fe{244} and lower coverage over the Fe{024} (Figure 3A). From Figure 5D, we see that the 24.12 and 25 V/nm‐cases reach equilibrium faster than the 20 V/nm case, indicating higher oxygen adsorption rates for higher EFs while their equilibrated oxygen coverage decreases, as observed experimentally (Figure 4A). Those models are consistent with EAP observation and translate the effect of EFs on partial pressure by polarizing and attracting gas molecules towards the surface^[20]^ (Eqn. S24). If the oxygen pressure increases, so does the oxygen partial pressure at the apex of the tip (Eqn. S24), which is consistent with literature and EAP observations.

Pressure enhancements at the apex of the tip for the 20, 24.12 and 25 V/nm EF correspond to 5.4×10^−5^, 2.7×10^−3^ and 6.7×10^−3^ mbar, respectively. The enhancement at the border of the field of view for 20, 24.12 and 25 V/nm EF are 3.9×10^−5^, 1.7×10^−3^ and 4.2×10^−3^ mbar, respectively. The pressure enhancement is not uniform due to dissipating electric field strength away from the tip of the hemisphere, as was obtained in our COMSOL simulation (see Figure S12B). Oxidation is a very fast process where at 24.12 V/nm (Figure 5B), we observe that the distribution starts at 5 s with distinct separated zones of oxygen coverage. At 10 s, we see the oxygen coverage pattern consistent with that observed at equilibrium, forming a model that is highly compatible with experimental data. This early development shows how the system is sensitive to initial conditions. Calculated oxygen coverages are higher than 0.6 ML while the oxygen coverages measured by EAP is ~0.3 ML. This deviation can be attributed to the out‐of‐equilibrium nature of the EAP approach ‐ constantly removing totally or partially the adsorbates from the surface. Ultimately, intense EFs as high as 36 V/nm completely prevent the interaction of the Fe(011) with oxygen if we do not adjust the O binding energies to take into account the formation of an oxide layer (Figure S18).

The theoretical EF results of 24.12 V/nm show close agreement with EAP observations. However, the theoretical model is also limited due to the difficulty of explicitly modeling subsurface oxygen for every facet. The experimental model also detects oxygen ions present in the system rather than oxygen coverage on the Fe grain, as the theoretical model does. Regardless of the limitations of the model, the correlation between the theoretical models and the experimental results gives us fundamental insights into the effects of electric fields on surface oxidation and how one can control the oxidation state of the metal in their presence. We also note that the inclusion of the fourth facet is crucial in the multi‐scale model. Without the inclusion of the {024} facet, one would not get the oxygen depletion zones around the Fe{024} (Figure S21) as seen experimentally. The inclusion of this fourth facet in the model was informed by experiment. Our work therefore demonstrates the critical need for a feedback loop between experiment and theory that enables the development of predictive models for such complex systems.

The influence of hydrogen on the oxidation mechanism has been observed in other systems, notably Mg^[51]^ or W^[52]^ and Fe,^[4]^ where intentionally added hydrogen is shown to promote field evaporation at lower applied EFs.[^4^, ^52^] In our case, no hydrogen nor water has been intentionally introduced in the APT system, but their presence is irrefutable and probably unavoidable. While it is established that H and H_2_O are naturally degassing from stainless steel walls of UHV chambers, this problem is intensified by the sensitivity of APT that consistently detects H in small quantities.[^51^, ^53^] Great efforts are currently underway within the atom probe tomography research community to mitigate background H and improve H analysis to (semi)quantify H natively found in materials by APT.^[54]^ In parallel, it will be necessary to include the influence of H and H_2_O in future models.

## Conclusion

In this work, we successfully demonstrated the capabilities of EAPs to observe Fe oxidation under high EFs in real‐time. EAP observations highlight the effects of surface structures, showing that Fe oxidation primarily occurs on open facets like Fe{244} and Fe{112}, while high‐coordination facets like Fe{024}, Fe{013} and Fe{011} remain relatively immune to the initial stages of oxidation. EAP experiments supported by DFT calculations and a mean‐field model on a multi‐faceted catalytic grain, combined with statistical‐mechanical considerations, lead to the same conclusion: intense EFs have strong influences on Fe oxidation by polarizing and attracting gas molecules towards the surface, increasing de facto the local pressure through a local electric field enhancement at the apex of the tip.[^20^, ^25^, ^26^, ^28^, ^50^, ^55^, ^56^] Excessive EFs mitigate Fe oxidation by the occurrence of field evaporation and a decreased oxygen binding strength to the surface as elucidated through our DFT‐based model.

Additionally, through simple modifications to modern APT systems, EAP can help facilitate unique experiments to provide fundamental insight into EF‐enhanced chemical reactions. Further development could lead to more accurate measurements of localized electric fields, which will be the subject of further studies. Due to the physical nature of those effects, it is reasonable to think that they can be extrapolated to a broad range of chemical systems and open a new perspective of how one can dynamically influence a given reaction of interest with EFs. Further studies will be conducted on other systems to determine if electric fields are not only a parameter to consider but perhaps a universal control that may act on an elementary chemical reaction step.

## Supporting Information

The authors have cited additional references within the Supporting Information^.[38,57—77]^

1

## Conflict of Interests

The authors declare no conflict of interest.

## Supporting information

As a service to our authors and readers, this journal provides supporting information supplied by the authors. Such materials are peer reviewed and may be re‐organized for online delivery, but are not copy‐edited or typeset. Technical support issues arising from supporting information (other than missing files) should be addressed to the authors.

Supporting Information

Supporting Information

Supporting Information

Supporting Information

Supporting Information

Supporting Information

Supporting Information

Supporting Information

Supporting Information

Supporting Information

Supporting Information

Supporting Information

## Data Availability

The data that support the findings of this study are available from the corresponding author upon reasonable request.
