# Supplementary figures and images for: Elucidating the Role of Electric Fields in Fe Oxidation via an Environmental Atom Probe
Source: Angew Chem Int Ed Engl. 2025 Mar 21;64(18):e202423434. doi: 10.1002/anie.202423434 (PMC12036815; doi:10.1002/anie.202423434)

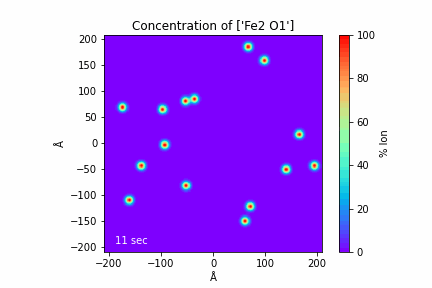

Supplement: Supplementary file 4 — Supporting Information [file ANIE-64-e202423434-s009.gif]

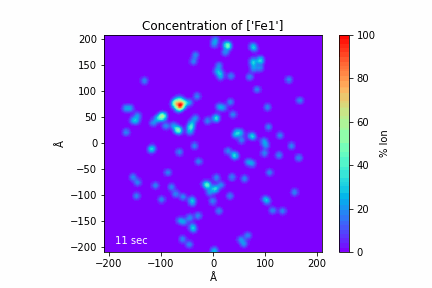

Supplement: Supplementary file 5 — Supporting Information [file ANIE-64-e202423434-s007.gif]

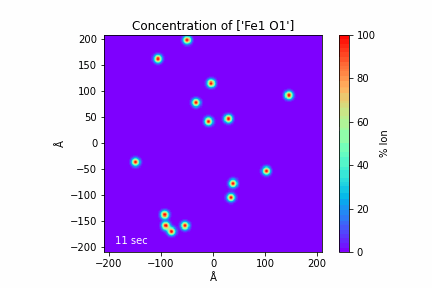

Supplement: Supplementary file 6 — Supporting Information [file ANIE-64-e202423434-s012.gif]

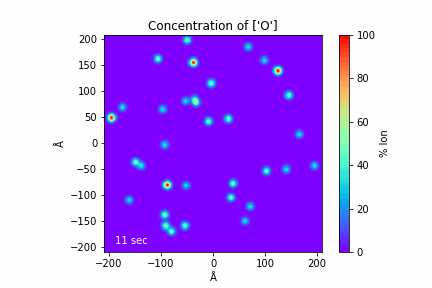

Supplement: Supplementary file 12 — Supporting Information [file ANIE-64-e202423434-s001.gif]
