# Supplementary material for: Elucidating the Role of Electric Fields in Fe Oxidation via an Environmental Atom Probe
Source: Angew Chem Int Ed Engl. 2025 Mar 21;64(18):e202423434. doi: 10.1002/anie.202423434 (PMC12036815; doi:10.1002/anie.202423434)
Supplement: Supplementary file 11 — Supporting Information [file ANIE-64-e202423434-s004.pdf]

Angewandte Chemie
Eine Zeitschrift der Gesellschaft Deutscher Chemiker

Supporting Information

Elucidating the Role of Electric Fields in Fe Oxidation via an Environmental Atom Probe

S. V. Lambeets, N. Cardwell, I. Onyango, M. G. Wirth, E. Vo, Y. Wang, P. Gaspard, C. F. Ivory, D. E Perea, T. Visart de Bocarmé, J.-S. McEwen**

Supporting information for

Elucidating the Role of Electric Fields in Fe Oxidation via an Environmental Atom Probe

Sten V. Lambeets,[a]*, Naseeha Cardwell,[b] Isaac Onyango,[b] Mark G. Wirth,[c] Eric Vo,[a] Yong Wang,[b,d] Pierre Gaspard,[e] Cornelius F. Ivory[b], Daniel E Perea,[c] Thierry Visart de Bocarmé,[e,f] Jean-Sabin McEwen[b,d,g]*

Dedication SVL, NC and IO equally contributed to the production of this publication.

[a] Dr. S.V. Lambeets, E. Vo
Physical and Computational Sciences Directorate
Pacific Northwest National Laboratory
902 Battelle Blvd, 99354, Richland WA, United States of America
E-mail: sten.lambeets@pnnl.gov, js.mcewen@wsu.edu

[b] N. Cardwell, Dr. I. Onyango, Prof. Y. Wang, Prof. C.F. Ivory, Prof. J.-S. McEwen
The Gene and Linda Voiland School of Chemical Engineering and Bioengineering
Washington State University
1505 Stadium Way, 99164, Pullman WA, United States of America

[c] M.G. Wirth, Dr. D.E. Perea
Environmental Molecular Sciences Laboratory
Pacific Northwest National Laboratory
902 Battelle Blvd, 99354, Richland WA, United States of America

[d] Prof. Y. Wang, Prof. J.-S. McEwen
Institute for Integrated Catalysis
Pacific Northwest National Laboratory
902 Battelle Blvd, 99354, Richland WA, United States of America

[e] Prof. P. Gaspard, Prof. Thierry Visart de Bocarmé
Centre for Nonlinear Phenomena and Complex Systems
Université Libre de Bruxelles
Campus Plaine CP231, B-1050, Brussels, Belgium

[f] Prof. Thierry Visart de Bocarmé
Chemistry of Surfaces, Interfaces and Nanomaterials
Université Libre de Bruxelles
Campus Plaine CP243, B-1050, Brussels, Belgium

[g] Prof. J.-S. McEwen
Departments of Physics and Astronomy/Chemistry/Biological Systems Engineering
Washington State University
99164, Pullman WA, United States of America

Supporting information for this article is given via a link at the end of the document.

1. Supporting information for experimental results

1.1 Methods

1.1.1 Fe specimen preparation

Fe specimens were prepared from a pure Fe wire (99.9%, Ø ~ 0.1 mm) by an electrochemical etching method, described elsewhere, using two different electrochemical etching steps. [33,34,47] The first etching stage uses a 25% perchloric acid (70%) in glacial acetic acid solution with 10-25 VDC. The second etching stage uses a 2% perchloric acid (70%) in 2-butoxyethanol with a 10-25 VDC voltage[47]. Prior to electrochemical etching, the Fe wire is cleaned by soaking it in pure phosphoric acid for 20 min. Fe presents a body-centered cubic (bcc) crystal system and systematically presents the same set of facets from sharpened wire. The specimen is cleaned by field evaporation with an initial APT analysis until that the surface presents the field evaporation of pure Fe – $^{54}Fe^{2+}$, $^{56}Fe^{2+}$, $^{57}Fe^{2+}$ and $^{58}Fe^{2+}$ at 27, 28, 28.5, and 29 Da, respectively. We also find a peak at 37 Da which compositionally is less than 1% of the ions collected. We attribute this peak to be $FeOH_2^{2+}$ likely coming from the surface resulting from the aqueous based electrochemical etching and/or from water found natively in the UHV system.

1.1.2 Field Ion Microscopy

FIM is an imaging technique that characterizes the surface structure of materials prepared as sharp needles with atomic lateral resolution (~0.2 nm). The images produced by FIM result from the ionization of imaging gas atoms, in our case Ne, over surface atoms of the positively biased substrate surface (i.e. Fe needle). This ionization is made possible by the application of a relatively intense external electric field (EF) on the surface. The presence of an EF as high as 35 V/nm is necessary to ionize Ne atoms and requires the specimen to be shaped as a sharp needle with few tens of nanometers radius curvature at the apex. Resulting positively charged Ne ions are projected toward a detector screen. The final FIM image gives a near stereographical projection of the apex on which surface facet structures can be identified by their Miller indices with the condition of having a relatively defect-free surface. In this work, the FIM images were obtained with a LEAP 4000X-HR Local Electrode Atom Probe System commercialized by CAMECA instruments. Data collection and analyses are performed using CAMECA's Integrated Visualization & Analysis Software (IVAS) version 3.8.8.

1.1.3 Atom Probe Tomography, field evaporation cleaning and electric field estimation

At 45 K, any addition of oxygen or attempt to observe a surface chemical reaction is problematic since oxygen is solid below about 54 K; oxygen is a liquid below about 90 K. At the same time, field evaporation is a thermally activated phenomenon, hence, the intensity of the electric field necessary to induce Fe field

evaporation decreases while the surface temperature increases. While the evaporation field of Fe is estimated as high than ~35 V/nm at 0 K, it is not the case for higher temperatures.[33,34,36–38] To estimate the field evaporation of Fe at 303 K, we proceed with an APT analysis of the Fe sample at 45 K and then at 303 K. We associate the applied voltage needed to field evaporate Fe field evaporation optimally at 45 K, to the value of 33 V/nm. The applied voltage has a linear relationship to the applied electric field and inversely linear to the radius curvature of the specimen: $V_{\text{applied voltage}} \propto \frac{F_{\text{field evaporation}}}{R_{\text{curvature}}}$.[3] Hence, if the radius curvature does not change significantly between the two measures, we can infer the value of the field evaporation at 303 K: $F_{303K} = \frac{F_{45K}V_{303K}}{V_{45K}}$.

After an initial APT analysis at 45 K (pulse ratio 10%, pulse frequency 100 kHz and 0.2% detection rate), the specimen is displaced away from the local electrode in a neutral position and the cryogenic system is turned off to let the system warm up to room temperature. The system is warmed up to 303 K which is the maximum temperature our system can endure. The temperature is maintained constant using the built-in heating system. At 45 K and prior to an experiment, the pressure of the APT system analysis chamber is $<2.6\times10^{-11}$ mbar, while at 303 K, the pressure is $<5.0\times10^{-11}$ mbar. A regular APT analysis is performed at 303 K to experimentally determine the evaporation field of Fe at this temperature using the same experimental parameters of pulse ratio and frequency, and 0.3% detection. Note that the indicated voltages are the addition of the static and pulsed voltage altogether. As shown on Table S1, the electric field required to field evaporate Fe is systematically lower at higher temperatures. Mass spectra of those APT analysis are found **Fig. S2A & B** and show that a significant amount of Fe^+ ions (~27%) are found at 303 K. Our experimental datasets enable us to estimate the evaporation field of Fe to 23.4 V/nm at 303 K, which is relatively close to the values found with Kingham plots reporting the relative mass/charge ratios of different element over the intensity of electric fields (between 20 and 21V/nm).[33,57,58] Additionally, we notice that the mass spectra at 303 K present a more important background noise which is associated to the fact that the cold finger is warm and does not trap the remaining contaminations in the UHV chamber. Those contaminants randomly hit the detector creating this amplified background noise. However, we do not notice a significant increase of the contaminants on the Fe surface. FeH_2O^{2+} (37 Da), has a similar relative presence towards Fe^{n+}, with 1 FeH_2O^{2+} ion for 100 Fe^{n+} detected at 45 K, and 1.5 FeH_2O^{2+} ion for 100 Fe^{n+} detected at 303 K.

Table S1: Specimen's radius curvature and their respective applied electric field to trigger field evaporation at 45 K and 303 K.

Specimen	$F_{0\,K}$ (V/nm)	$V_{45\,K}$ (V)	$F_{45\,K}$ (V/nm)	V_{303K} (V)	F_{303K} (V/nm) calculated
1	35	4200	33	3080	24.2
2	35	5610	33	3960	23.3
3	35	3037	33	2200	23.9
4	35	5445	33	3685	22.3

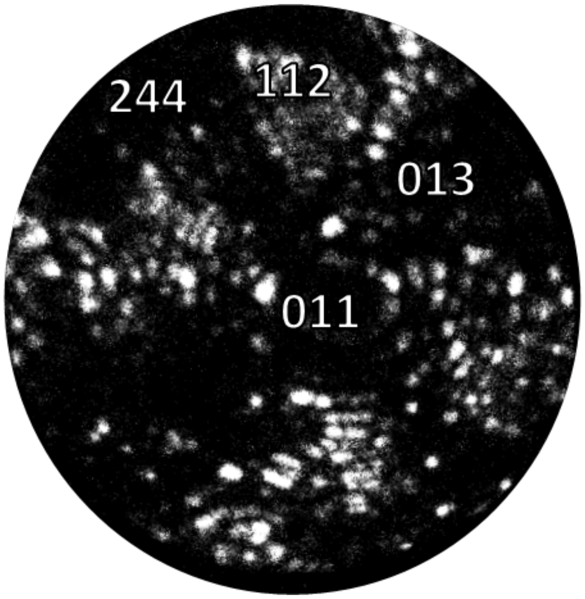

Figure S1: Experimental FIM of a (011)-oriented Fe needle specimen. Conditions of acquisition: 45.0 K, 3300 V (~35 V/nm), P(Ne) = 2.2×10^{-6} mbar. Main facets identified are Fe{112}, Fe{013}, Fe{244} and a central Fe(011).

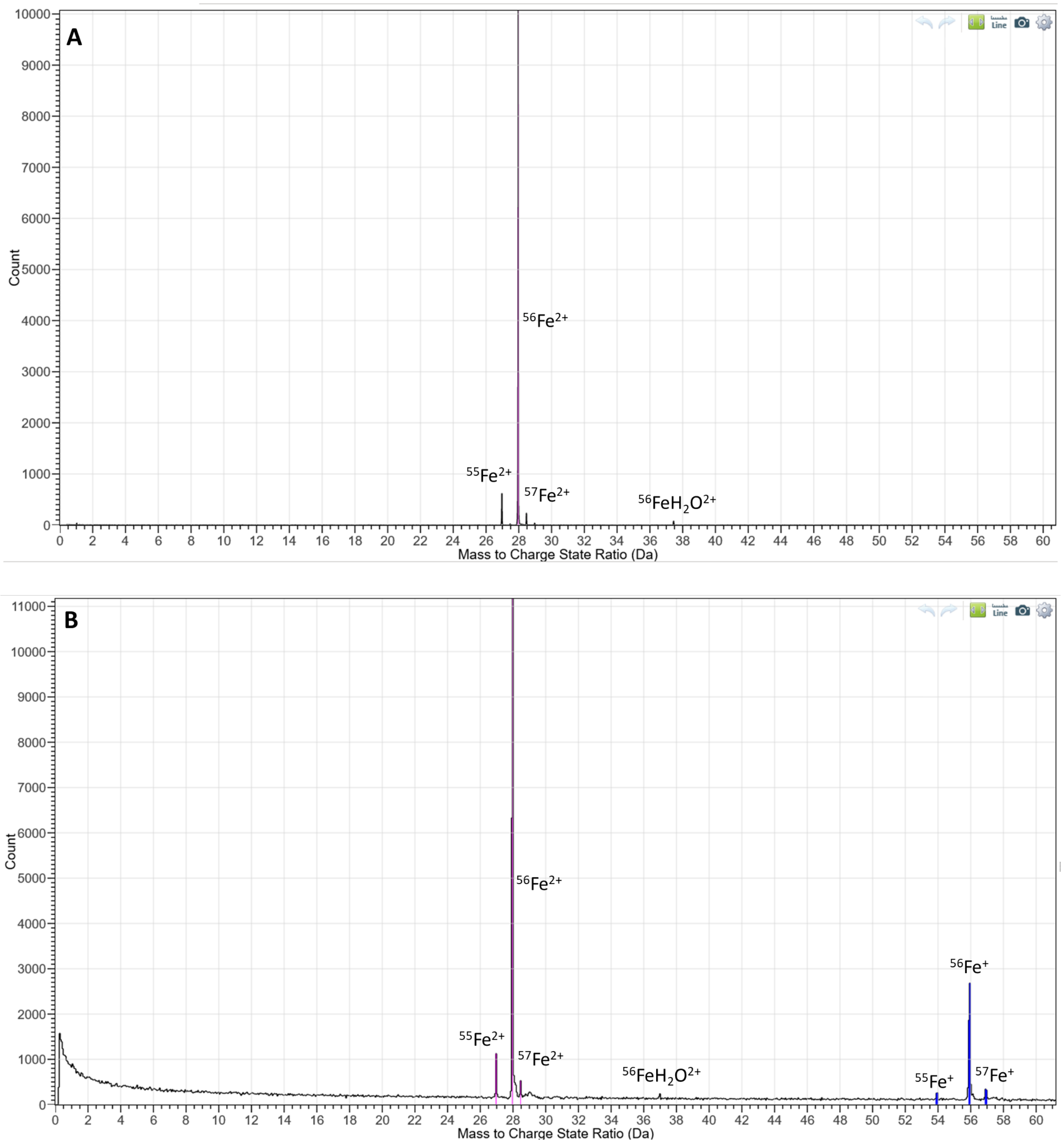

Figure S2: Mass spectra of the Fe specimen after the field evaporation cleaning at **(A)** APT experiment at 45 K finishing at ~5200V (static+pulse) (10% pulse fraction, 125kHz pulse frequency and 0.2% detection rate target with an estimation of 0.002% detection associated to the background noise) and **(B)** APT experiment at 303 K (static+pulse) finishing at ~3100V (10% pulse fraction, 125kHz pulse frequency and 0.3% detection rate target with an estimation of 0.02% detection associated to the background noise).

1.1 Complementary results to the Section 2.3.2. Spatial-time conversion and constant electric field

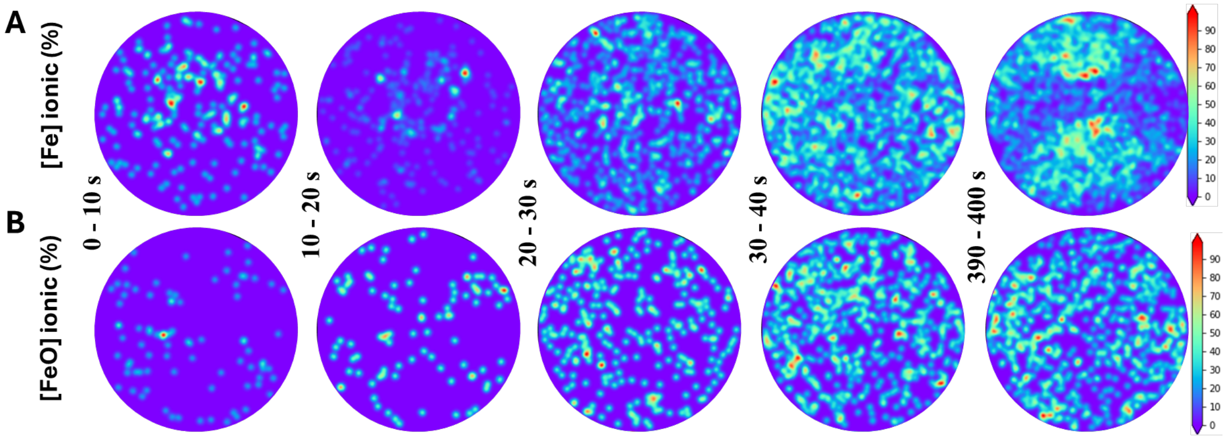

Figure S3: 2D projection composition maps of **(a)** $Fe^{n+}(\%)_{ionic}$, **(b)** $FeO^{n+}{}_{ionic}$ of a Fe specimen exposed to 1.2×10^{-7} mbar of pure O_2 with a constant 18 V/nm (23.4 V/nm pulsed) applied EF.

1.2 Notes on the EAP measurement of Fe surface composition while exposed to oxygen.

As can be seen from **Figure 4**, there is a direct correlation between the applied voltage and the apparent composition. We note that we must be attentive to the eventuality of oxygen accumulation over time. With a 1.2×10^{-9} mbar exposure to oxygen over nearly 3 hours of experiment, the surface of interest is exposed to an equivalent of ~10 Langmuir. Despite those exposures, we observe that the oxygen rate of the surface with one applied EF does not depend on its previous applied EF, suggesting that oxygen does not accumulate over time on the surface, and that we are effectively field evaporating the majority, if not the entire amount of oxygen on the surface.

The apparent enriched oxidation at low EFs can be associated to two separate or combined phenomena. The first is that the EF directly impacts the surface dissociative adsorption of O_2 over Fe. The second is the possibility of facing "differential field evaporation" phenomena. Fe oxides require a lower EF for field evaporation as compared to pure Fe. Subsequently, EFs such as between 20 and 25 V/nm are needed to trigger the field evaporation of Fe oxides. From these observations comes the challenge to identify the source of this correlation.

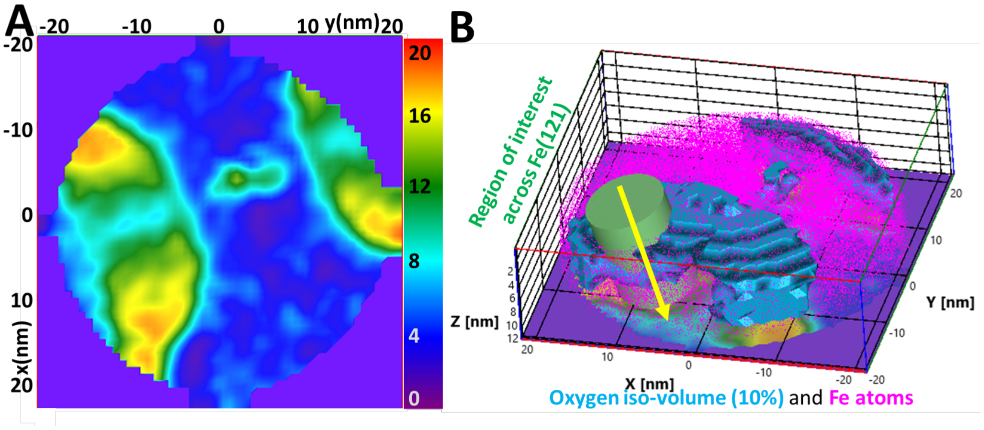

Figure S4: Influence of the applied EF on the quantity of oxygen atoms collected by APT after the exposure of O_2 (1.2×10^{-9} mbar) for 30 min at +25 V/nm. **(a)** 2D O (%) composition heat map. **(b)** APT reconstruction of a Fe specimen after exposure. Purple dots correspond to detected Fe^{n+}. Iso-composition surfaces encapsulating the regions greater than 10% O atoms are shown as blue volumes whose positions reflect the Fe{112} facets. The green cylinder of 10 nm diameter is used to plot the 1D concentration profile along the direction pointed by the yellow arrow.

2. Supporting Information for Computational Work

2.1 DFT Calculations

2.1.1 Calculation Details

Density Functional Theory (DFT) calculations were performed using the Vienna *Ab initio* Simulation Package (VASP)[59,60]. The Projector-Augmented Wave (PAW) method[61,62] was used with a planewave basis set and energy cutoff of 400 eV. Electron smearing was modeled using the Methfessel-Paxton method[63] with a smearing width of 0.1 eV. The electronic energetic and ionic force relaxations tolerances were set at $1.0\cdot10^{-4}$ eV and $3.0\cdot10^{-2}\,\frac{\text{eV}}{\text{Å}}$, respectively, where the conjugate gradient method was used for ionic relaxations. The Revised Perdew-Burke-Ernzerhof (RPBE) method was used to model the electron exchange and correlation[64,65] with PAW potentials for Fe and O released from Sept 2000 and Apr 2002, respectively. A Monkhorst-Pack **k**-point grid[66] of $4\times4\times1$ was used to sample the first Brillouin zone for all calculations, except for gas-phase molecular oxygen in which the gamma point was used. Spin polarization was included to account for the magnetic moment of each system. The lattice constant for body-centered-cubic (bcc) Fe was found to be 2.866 Å (experimentally 2.87 Å[67]) using a 20×20×20 Gamma point centered k-point grid.

2.1.2 Facet Modeling and Oxygen Adsorption Sites

Four bcc Fe facets were investigated for this work—Fe(100), Fe(110), Fe(111), and Fe(024), where the fourth facet, Fe(024) was added to the investigation due to the experimentalists determining it to be of particular interest to the study. Each facet was modeled using a (2×2) supercell, with Fe(100) and Fe(110) containing four layers, Fe(111) containing 6 layers, and Fe(024) containing 11 layers, where the top two (for Fe(100) and Fe(110)), three (Fe(111)) and five (Fe(024)) layers were allowed to relax. Previous work determined the most favorable adsorption site for oxygen at the lowest coverage for each facet (besides Fe(024), which we tested) (**Figure S5**)[20], where, for this investigation, the calculations were performed with oxygen at the most favorable adsorption site for each respective Fe facet across the full coverage range.

For the Fe oxide calculations, the Fe(001) facet was chosen as a representative surface. A slab model consisting of six layers was constructed, with the top layer of Fe atoms replaced with oxygen atoms, following a similar methodology of Ossowski and Kiejna[68]. The calculations were converged using the same DFT parameters described above, with the bottom three layers fixed in place. The vacuum layer was increased to 1.4 nm. Oxygen was adsorbed in the same favorable position as on Fe(001). This new configuration is referred to as FeO(001) moving forward.

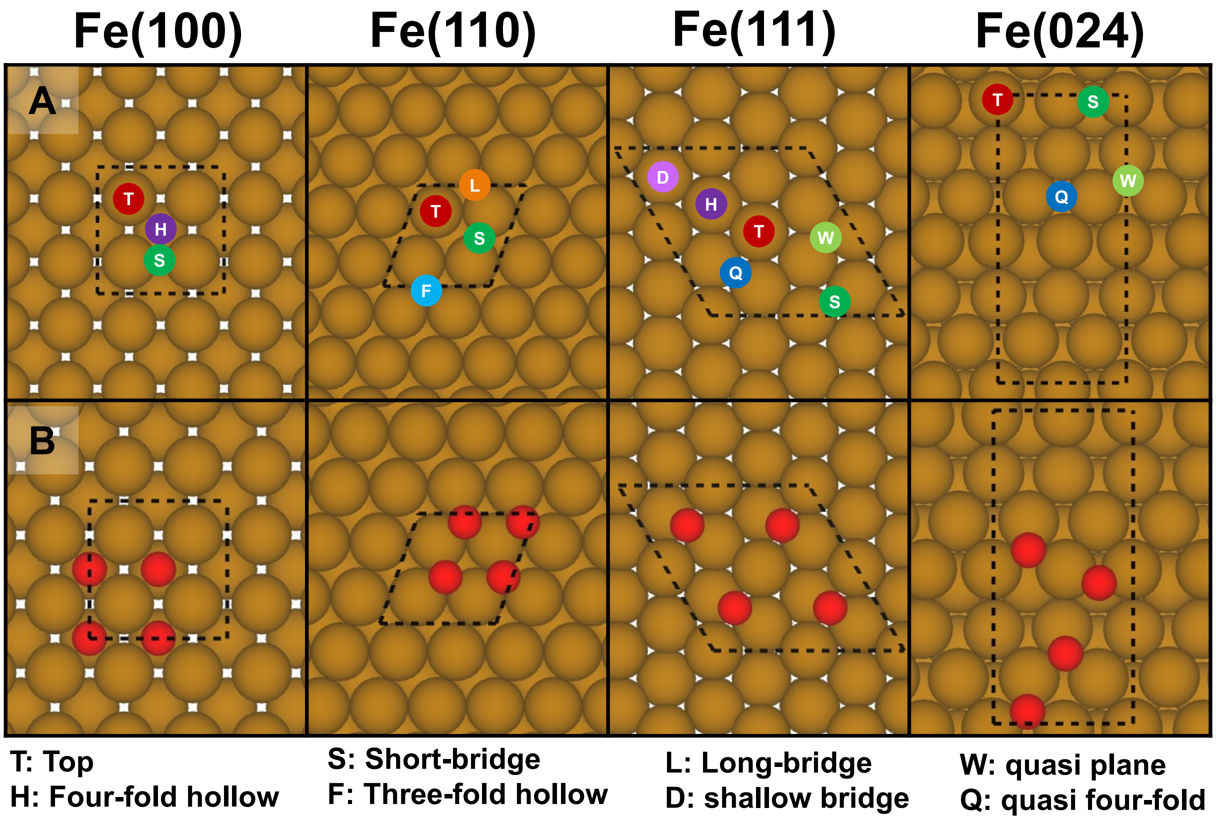

Figure S5: A Possible and tested adsorption sites for oxygen on each Fe facet. **B** The most favorable sites are the four-fold hollow, three-fold hollow, quasi-four-fold hollow sites for Fe(100), Fe(110), and Fe(111), respectively. Fe(024) has multiple favorable sites depending on the number of adsorbed oxygens (quasi-four-fold/quasi plane/short-bridge hollow), which is detailed in **Figure S5**. On the (024) surface, we define a coverage of 1 ML where all quasi plane sites would be occupied, which would necessitate the adsorption of four oxygen species. The red circles are oxygen and brown Fe.

The oxygen coverage was varied from 0.25-1.00 monolayer [ML] in increments of 0.25 ML, where coverage is defined as the ratio of adsorbed oxygen atoms to surface Fe atoms (e.g., 1 ML = 4 oxygen atoms/4 Fe atoms). **Figure S6** shows each system of adsorbed oxygen on the respective Fe facet for the full oxygen coverage range. Note that for Fe(024), the adsorption site for oxygen changes with an increasing number of oxygen atoms; this is due to the interacting molecules changing the preferred adsorption site for oxygen on Fe(024), however, the co-adsorbed oxygen atoms' distances from one another are still of the same magnitude as the other facets and were found to have a negligible effect on the calculated adsorption energies.

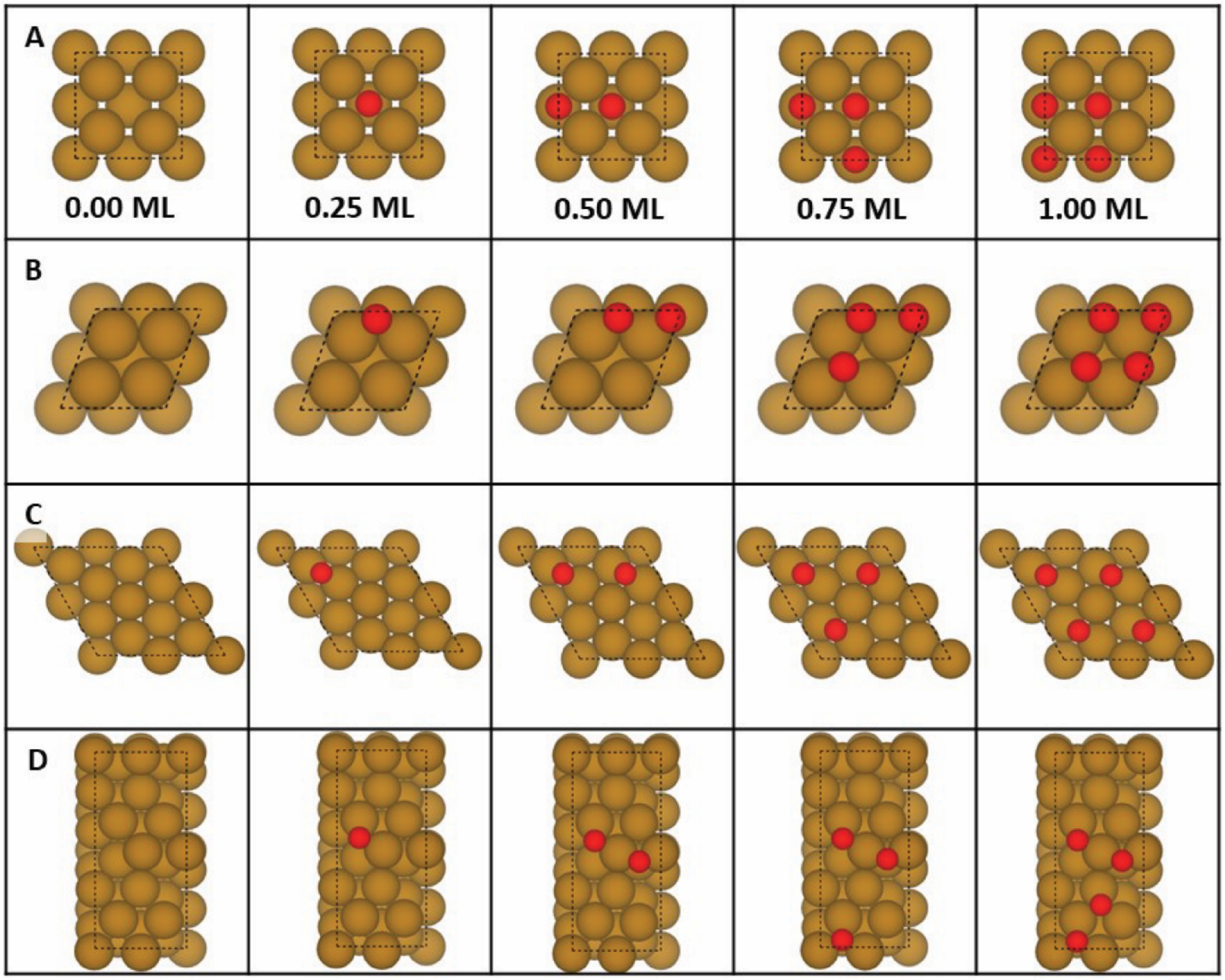

Figure S6: Visual representation of the increasing coverage on **A** Fe(100), **B** Fe(110), **C** Fe(111), and **D** Fe(024) at the most favorable adsorption sites. The red circles are oxygen and brown Fe. The coverage increases from 0.00 ML to 1.00 ML in increments of 0.25 ML. The red circles are oxygen and brown Fe.

2.1.3 Simulating the Electric Field

Here, the electric field tested ranged from +20.0 V/nm to -10.0 V/nm in increments of 2.5 V/nm for each oxygen-Fe facet system. Neugebauer and Scheffler proposed a method to simulate a uniform electric field within DFT calculations, used here to model the application of an EF[69]. This method introduces a dipole sheet in the middle of the vacuum region to cancel out the electrostatic field in the vacuum regions between the periodic slabs within the calculation. Feibelman et al.[70] noted that the vacuum width must be large enough to prevent field emission of the electrons into the vacuum which would cause poor convergence of the charge density. However, to minimize the computational cost, the vacuum width should not be too small. A too-small vacuum width would also cause complications in the calculations as well. We performed preliminary calculations to determine which vacuum width to use, shown in the next section.

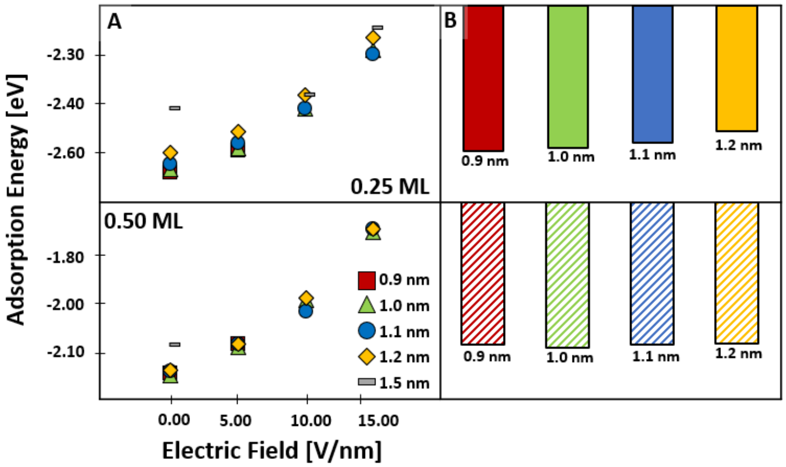

Figure S7: A Calculated adsorption energies of oxygen on Fe(110) at 0.25 ML and 0.50 ML, with different tested vacuum widths (see legend) with electric fields ranging from 0.0 to +15.0 V/nm in increments of 5.0 V/nm. Any missing data is indicative of a calculation that would not converge. **B** Comparison of the adsorption energies at 0.25 ML and 0.50 ML at a 5 V/nm field, showing that the energies for 0.9-1.2 nm are similar to one another. From here, a vacuum width of 1.0 nm was chosen for the succeeding calculations.

2.1.4 Vacuum Test

An investigation into the adsorption energy of oxygen on Fe (referred to as O/Fe moving forward), as it depends on the catalyst system vacuum width, was conducted to determine the optimal vacuum width for the succeeding calculations. Calculations of the adsorption energy of O/Fe at 0.25 ML and 0.50 ML for Fe(110) at various electric fields were performed (**Figure S7**) with vacuum widths of 0.9 nm, 1.0 nm, 1.1 nm, 1.2 nm, and 1.5 nm, where it was found that there was a negligible difference in oxygen adsorption energy at both coverages between 0.9 and 1.2 nm (< 0.02 eV). From this, a vacuum width of 1.0 nm was chosen for all subsequent calculations for all catalyst systems.

2.1.5 Adsorption Energy

The adsorption energy, E_{ads} [eV/molecule] of the O/Fe systems was modeled using the mean-field approach, which assumes that the adsorbing molecules (here, molecular oxygen) can be described as an analytical function of coverage[71,72]. This allows us to combine the adsorbate-adsorbate lateral interactions as a function of coverage, thus simplifying the model. The adsorption energy, E_{ads} [eV], was calculated according to the following equation:

$$E_{ads} = \frac{E_{sys} - E_{Fe} - N \cdot \frac{1}{2} E_{O_2}}{N} \qquad \text{(Eqn. S1)}$$

where E_{sys} [eV] is the energy of the O/Fe catalyst system, E_{Fe} [eV] is the energy of the respective Fe catalyst without any oxygen adsorbed, N is the number of adsorbed oxygen atoms (ranging from 1-4), and E_{O_2} is the energy [eV] of the gas-phase spin-polarized molecular oxygen. The adsorption energy of O/Fe was calculated for all four Fe facets at the four different oxygen coverages for the full electric field range (**Figure 1**). See the main text for the analysis of this data.

The calculated adsorption energies for oxygen on Fe, using E_{O_2} as the energy [eV] of the gas-phase atomic oxygen (and thus the $\frac{1}{2}$ multiplier not being included), closely aligns with those reported by Xu et al.[73] (-5.69 eV and -5.46 eV for our values and the literature values, respectively, for 0.25 ML on Fe(110) in the absence of an electric field). Despite the RPBE functional's known tendency to over-predict adsorption energies, this close agreement suggests that our results are consistent with the expected range for this functional.

2.1.6 Oxygen Adsorption on an Fe Oxide

As we have found that the experimental system already contains Fe oxides at the beginning of the experiment, it is important to consider this presence of oxides when developing the computational model. We created an FeO(001) facet and adsorbed oxygen on the surface in a step-wise manner (**Figure S8A**) to test how the presence of an oxide affects the adsorption of atomic oxygen. We found that the presence of an oxide decreases the overall adsorption energy of oxygen. At the lowest coverage, this difference is pronounced (**Figure S8B**), where the adsorption energy of oxygen on FeO(001) is approximately 2.43 times smaller than that of Fe(001)—the energy difference is about 1.49 eV between the two. This difference decreases as the coverage increases, with the adsorption energy of FeO(001) remaining smaller than that of Fe(001) at all coverages. The adsorption energy also increases with increasing coverage, which is a different trend than the non-oxide Fe facets, where the adsorption energy decreases with increasing coverage. Although not explicitly explored, since it is out of the scope of this investigation, we hypothesize that this increase in adsorption energy with increasing coverage is due to an increase in the stability of the oxide layer. This means that the presence of an oxide, even just one layer, can greatly decrease the overall oxygen coverage over the entire Fe grain, and thus would impact the corresponding surface plot models. Additionally, if we compare the location of the oxide layer between the different coverage systems, we see that the adsorption of atomic oxygen pushes the oxide layer further down into the Fe slab.

We applied electric fields to the 0.25 ML case for FeO(001), as well (**Figure S9A**), finding that the FeO(001) system still has a parabolic dependence on the electric field at a coverage of 0.25 ML (**Figure S9B**). The overall energies, again, are much lower (less negative) for the oxide system compared to the non-oxide system.

Although the trends we have discovered for the adsorption of oxygen on an FeO(001) facet should still hold true, we have found that FeO systems are best described as antiferromagnetic (AFM) rather than magnetic, which is how they are treated for the calculation results used in **Figures S8** and **S9**. When applying AFM ordering to the systems, we found that the adsorption of oxygen is even lower, and slightly endothermic, with a value of approximately 0.18 eV (**Figure S10**).

Still, since we found that the presence of an oxide layer greatly impacts the adsorption of oxygen, we have incorporated this decrease in energy to the corresponding surface plot models.

A

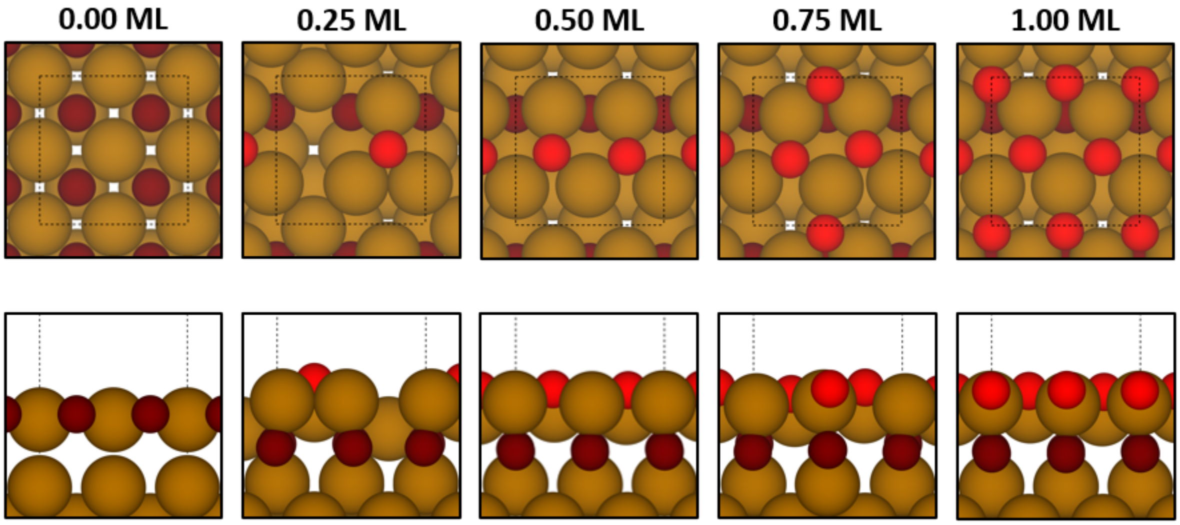

B

Coverage [ML]	E_{ads} [eV]	Difference	Fe/FeO E_{ads}
**0.25**	-1.04	1.49	2.43
**0.50**	-1.34	1.13	1.84
**0.75**	-1.74	0.54	1.31
**1.00**	-1.81	0.38	1.21

Figure S8: A. Visual representation of the adsorption of oxygen over FeO(001) at increasing coverages with the **B.** Adsorption energy, difference and ratio in energies between the oxidized and non-oxidized Fe(001) systems. The brown, dark red, and red circles are Fe, oxygen in the oxide, and adsorbed oxygen, respectfully.

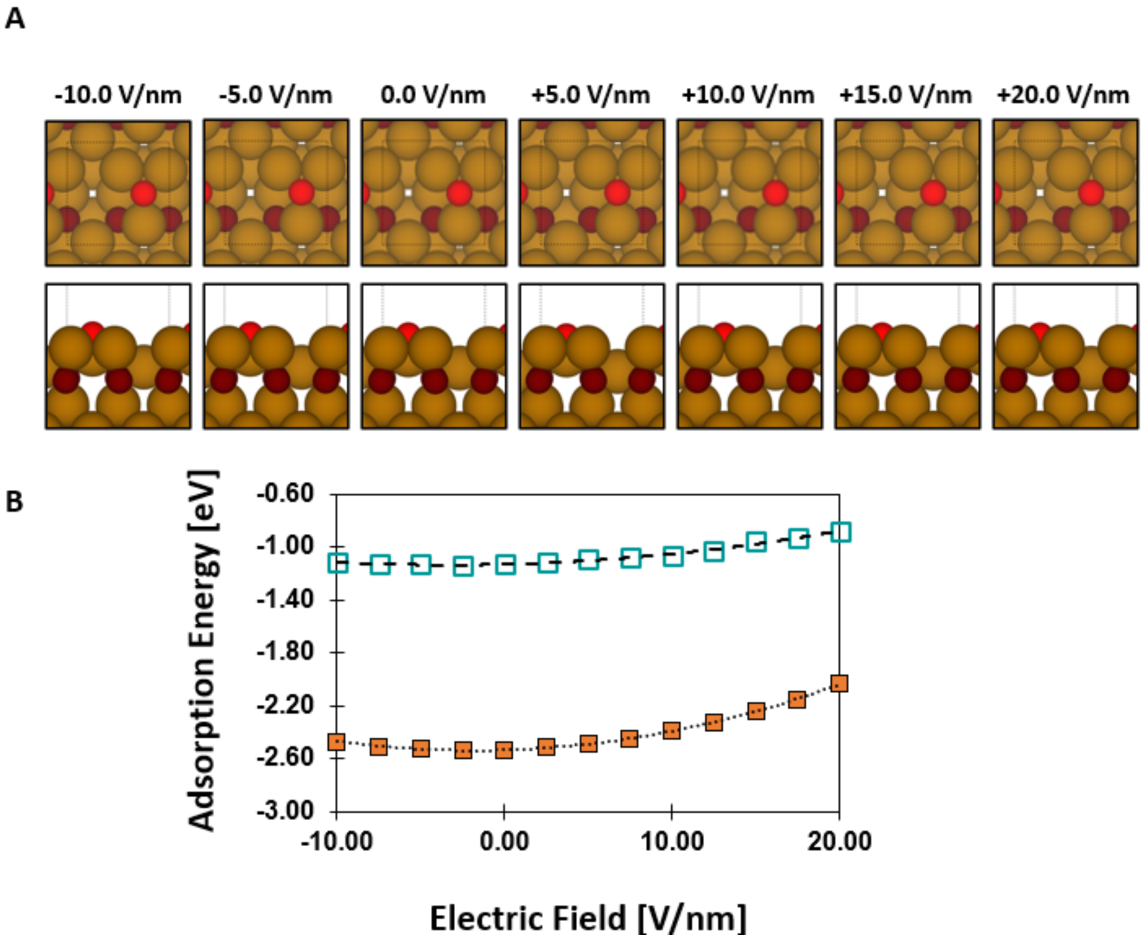

Figure S9: A. Visual representation of the adsorption of oxygen on FeO(001) at different externally applied electric field values. The brown, dark red, and red circles are Fe, oxygen in the oxide, and adsorbed oxygen, respectfully. **B.** Calculated adsorption energies of oxygen on FeO(001) and Fe(001) as a function of electric field, where the electric field was varied from -0.10 V/nm to +0.2 V/nm in increments of 0.025 V/nm. The open teal squares represent FeO(001) and the closed orange squares represent Fe(001).

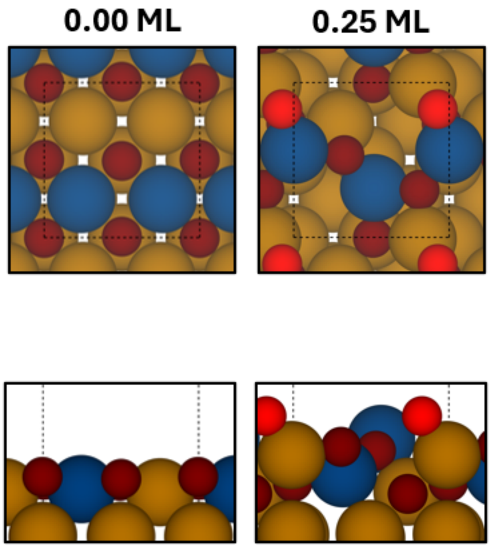

Figure S10: Visual representation of the adsorption of oxygen over FeO(001) at both 0.00 ML and 0.25 ML, using antiferromagnetic ordering. The brown, dark blue, dark red, and red circles are spin up Fe, spin down Fe, oxygen in the oxide, and adsorbed oxygen, respectfully.

2.1.7 Mean-Field Adsorption Energy Error Analysis

The Root Mean Square Error (RMSE) for all datapoints was calculated using the equation:

$$\mathrm{RMSE} = \left(\frac{\left(\sum_{N} E_{ads}(\theta_i) - \hat{E}_{ads}(\theta_i)\right)^2}{N}\right)^{\frac{1}{2}} \qquad \text{(Eqn. S2)}$$

where $E_{ads}(\theta_i)$ is the calculated adsorption energy and $\hat{E}_{ads}(\theta_i)$ is the mean-field estimated adsorption energy at the ith electric field value. The RMSE determines the deviation of the datapoints from the analytical model (here, a second-order parabola), with a lower RMSE value indicating a better fit of the data to the model. The largest RMSE using this equation is 51 meV for the O/Fe(111) at 0.50 ML indicating that the mean-field approach accurately captures the field-dependent O/Fe adsorption energy regardless of facet-type or oxygen coverage.

Table S2: Root Mean Square Error (RMSE) [meV] analysis of the calculated adsorption data on all facets.

Coverage	Fe(100)	Fe(110)	Fe(111)	Fe(024)
0.25 ML	2.76	3.30	4.69	18.87
0.50 ML	3.89	11.52	51.47	12.57
0.75 ML	1.94	1.58	4.59	3.94
1.00 ML	2.27	1.72	3.04	23.10

2.2 Modeling the Fe Grain as a Parabolic Field Emitter Tip

For comparison to the experimental results, the theoretical results approximated the multi-faceted Fe grain as a field emitter tip, initially modeled as a paraboloid, following the methods from Bray et al.[8,20] (**Figure S11A**). After analysis, it was found that a more realistic representation of the tip uses a hemispherical geometry (**Figure S11B**). The following equations are for a hemisphere geometry.

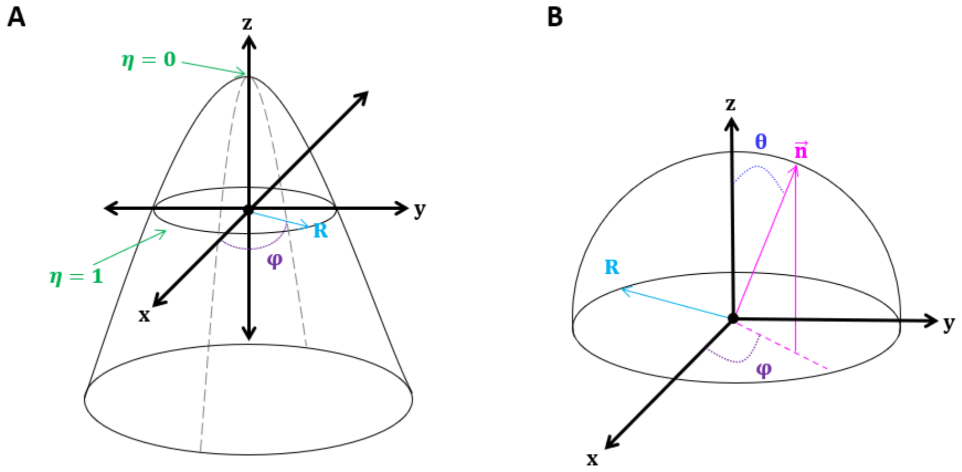

Figure S11: A. Parabolic model of the field emitter tip with the surface represented in parabolic coordinates, and **B.** hemispherical model of the field emitter tip in spherical coordinates.

The grain model for a hemisphere is:

$$z = \sqrt{R^2 - x^2 - y^2} \qquad \text{(Eqn. S3)}$$

where R is the radius of the hemisphere. From **Figure 11**, we note that x, y, z are the coordinates along the x, y, z -directions, ϕ is the azimuth angle from the x-axis in the xy-plane (ranging from 0 to 2π), θ is the

zenith angle (ranging from 0 to $\frac{\pi}{2}$ for a hemisphere) , defined by the unit normal vector, $\vec{n}$, and that the projection of $\vec{n}$ defines ϕ with respect to the x-axis.

Converting the cartesian coordinates (x, y, z) to their spherical counterparts (r, θ, ϕ) with $r = R$ gives:

$$x = R\sin(\theta)\cos(\phi) \quad \text{(Eqn. S4)}$$
$$y = R\sin(\theta)\sin(\phi) \quad \text{(Eqn. S5)}$$
$$z = R\cos(\theta) \quad \text{(Eqn. S6)}$$

where $\phi = \arctan\left(\frac{y}{x}\right)$.

We can define $\vec{n}$ in Cartesian coordinates, as well:

$$\vec{n} = \left(n_x, n_y, n_z\right) = (\sin\theta\cos\phi, \sin\theta\sin\phi, \cos\theta) \quad \text{(Eqn. S7)}$$

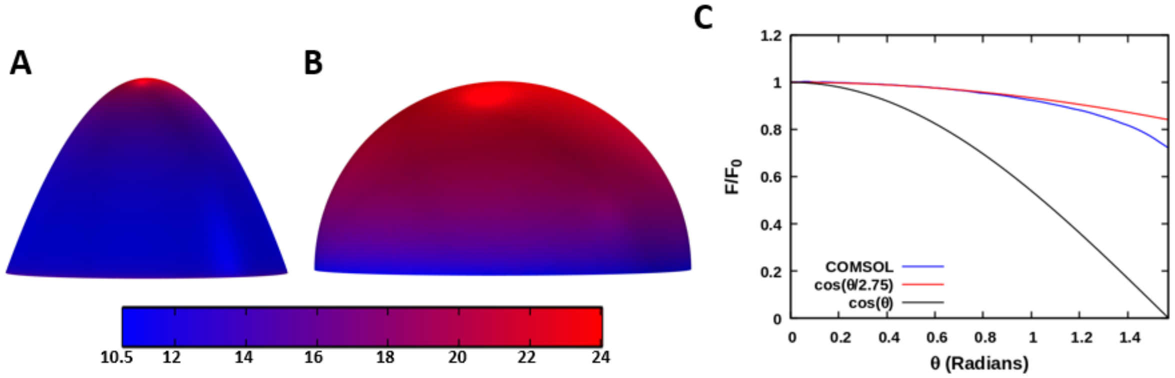

Figure S12: Schematic of the distribution of electric field on a **A.** parabolic tip and **B.** hemispherical tip. **C.** Change of electric field with varying zenith angle θ of the hemispherical (blue curve) and parabolic (black curve) tips for a field of view of $0 - \frac{\pi}{2}$. Also shown is an approximation for the electric field dependence for a hemispherical tip (red curve). In **A.** and **B.**, the legend refers to the intensity of the electric field, where a deep blue coloring represents a less intense field, and a deep red coloring represents a more intense field.

Using COMSOL, simulations were conducted to analyze the electric field distribution on a hemispherical tip. Comparing these simulations with cosine functions of the zenith angle revealed that the spatial distribution of the electric field on the hemispherical tip within a 0 - 35° can be represented by

$$F = F_0 \cos\left(\frac{\theta}{2.75}\right) \quad \text{(Eqn. S8)}$$

With this function, the electric field is ~2% lower at the border of the field of view, compared to a decrease of ~15% for a paraboloidal tip. The details for how the COMSOL models were created can be found in the following subsection.

2.2.1 COMSOL model details

COMSOL Multiphysics® v6.2 is a commercial finite element software package that allows the simultaneous simulation of multiple, coupled transport equations in a geometry assembled in 0-, 1-, 2- or 3-dimensions. Our 3D model was assembled in COMSOL by choosing a coordinate system, and then fabricating a geometry to which we apply our model equation and boundary conditions.

The Poisson equation with zero space charge density, ρ_e=0, and constant dielectric permittivity is selected from COMSOL's Electrostatics physics menu,

$$-\nabla \cdot (\varepsilon_r \varepsilon_0 \nabla \phi) = \rho_e \quad \text{(Eqn. S9)}$$

to calculate the electric potential, ϕ, inside of the evacuated container. Here ε_r=1 is the relative permittivity and ε_0 is the permittivity of vacuum. The emitter tip is a parabola rotated 360° about its z-axis which has a radius of curvature at the apex, R_c= 20 nm, with a base radius of 55 nm and a height of about 80 nm, mounted on a cylinder with a 55 nm radius and a height of 200 nm (**Figure S13**). This emitter tip is mounted in the center of the floor of a cylindrical container with a diameter of 10 μm and a height of 20 μm. A geometric fillet with radius R_c is used to smooth the transition from the paraboloidal tip to the cylindrical base. The upper surface of the container is held at ground while the emitter and cylindrical base are held at +1120 V to produce an apex electric field of about 24 V/nm. The remaining container surfaces are insulated.

The triangular mesh elements on the surface of the emitter tip have a maximum edge length of R_c/20 and on the cylindrical base, R_c/10, eventually yielding a mesh count of 14.6 MM tetrahedral elements throughout the container volume. A stationary, i.e., steady-state, default solver produces a matrix with 19.6 MM degrees of freedom which converges rapidly and solves for the electric potential in about 6 minutes. For the hemispherical emitter tip, a hemisphere with radius, R_c, is placed on top of a cylindrical base and the voltage is adjusted to +710 V to produce an apex electric field of about 24 V/nm.

Figure S14 shows representative isopotential and electric field lines for both the paraboloidal and hemispherical emitter tips. **Figure S12** shows the surface normal electric field strength on each of these

tips. Note that this model was posed in a way that would produce a symmetric result about the z-axis and could have been solved in a 2D format which would use far less computational resources to produce the same answer. However, when geometric asymmetries or symmetry-breaking is included in the calculations, the 3D format will be required.

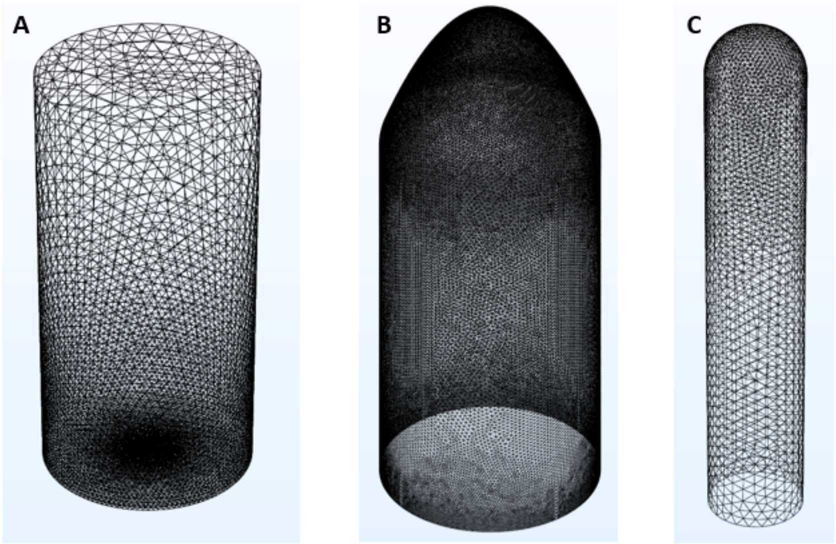

Figure S13: A. Geometry and tetrahedral mesh used in the volume of the probe container. **B.** Geometry and triangular surface mesh used on the paraboloidal probe. **C.** Geometry and triangular surface mesh used on the hemispherical probe.

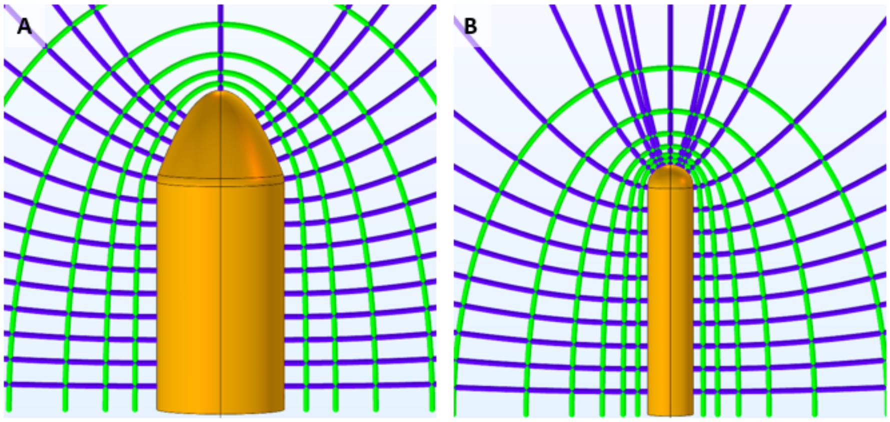

Figure S14: Isopotential lines (green) and electric field lines (blue) for **A.** the paraboloidal emitter tip and **B.** the hemispherical emitter tip.

2.3 Kubic Harmonic Models

For comparison to experimental efforts, the Fe facets were modeled as one multi-faceted catalytic Fe grain, approximated as a hemispherical field emitter tip, following a similar methodology as McEwen et al. [8,11], allowing for the projection of the bcc lattice onto the catalyst grain and the location of vital facets to be noted. Here, Fe(110) is located at the apex of the tip, and the subsequent facets located elsewhere, shown in **Figure 1**. This allows for the determination of the behavior of the grain as a whole and enables the consideration of the anisotropy of a realistic catalytic grain in which the various facets behave differently from one another due to external factors, such as applied electric fields, temperature, and oxygen partial pressure.

Using this model allows us to then expand the O/Fe kinetic constants as they depend on the orientation of the grain surface using Kubic Harmonics (KH)[43] which are non-reducible polynomial functions of the unit normal vector $\mathbf{n} = (x, y, z)$ that defines the grain surface orientation. This unit normal vector is defined with respect to the lattice vectors of the underlying bcc Fe crystal. Turning this into spherical coordinates gives the equations S4-S6. This allows for the condition: $x^2 + y^2 + z^2 = 1$ (Cond. 1) to be met. This also allows for a relation between the three cartesian coordinates of the unit normal vector to the Miller indices (hkl) of the respective Fe facets:

$$\mathbf{n} = (x, y, z) = \frac{(h, k, l)}{\sqrt{h^2 + k^2 + l^2}} \qquad \text{(Eqn. S10)}$$

From here, functions that are invariant under the grain symmetry operations must be determined. The functions must be polynomials of the coordinates, (x, y, z), that are invariant under the following transformations:

$$x \rightarrow -x \qquad \text{(Cond. 2)}$$

$$y \rightarrow -y \qquad \text{(Cond. 3)}$$

$$z \rightarrow -z \qquad \text{(Cond. 4)}$$

$$x \leftrightarrow y \qquad \text{(Cond. 5)}$$

$$y \leftrightarrow z \qquad \text{(Cond. 6)}$$

$$z \leftrightarrow x \qquad \text{(Cond. 7)}$$

$$x \rightarrow y \rightarrow z \rightarrow x \qquad \text{(Cond. 8)}$$

Due to the first three conditions (Cond. 2-4), these polynomials must be even powers of (x, y, z). This specific invariance condition is due to the "type" of irreducible operation which here is the type α. For each "type" there are sets of KH (functions that transform into one another via the symmetry operations). For this "type," and up to degree-8 (inclusion of data from four facets), we obtain the following four irreducible functions:

$$1 \quad \text{(Eqn. S11)}$$
$$x^4 + y^4 + z^4 \quad \text{(Eqn. S12)}$$
$$x^2y^2z^2 \quad \text{(Eqn. S13)}$$
$$x^8 + y^8 + z^8 \quad \text{(Eqn. S14)}$$

This means that for a single facet, a certain parameter (e.g., desorption energy of oxygen, E_{dO}) can be expanded via KH using an additive combination of the aforementioned irreducible functions like so:

$$E_{dO} = E_{dO}^{(0)}(1) + E_{dO}^{(4)}(x^4 + y^4 + z^4) + E_{dO}^{(6)}(x^2y^2z^2) + E_{dO}^{(8)}(x^8 + y^8 + z^8) \quad \text{(Eqn. S15)}$$

where the coefficients, $E_{dO}^{(0)}$, $E_{dO}^{(4)}$, $E_{dO}^{(6)}$, and $E_{dO}^{(8)}$ can be obtained for each facet separately by fitting data to this equation (see next section for details).

However, this expansion assumes that the grain is centered around the (001) facet. To compare to experimental work, our model needs to be centered around the (011) facet, so a simple rotation of the grain must be done:

$$x \rightarrow x \quad \text{(Cond. 9)}$$
$$y \rightarrow \frac{y - z}{\sqrt{2}} \quad \text{(Cond. 10)}$$
$$z \rightarrow \frac{y+z}{\sqrt{2}} \quad \text{(Cond. 11)}$$

This gives the new expansion equation that is used for the kinetic equations:

$$E_{dO} = E_{dO}^{(0)}(1) + E_{dO}^{(4)}\left(x^4 + \frac{1}{2}y^4 + 3y^2z^2 + \frac{1}{2}z^4\right) + E_{dO}^{(6)}\left(x^2\frac{(y^2 - z^2)^2}{4}\right) + E_{dO}^{(8)}(x^8 + \frac{1}{8}\left(y^8 + z^8 + 70y^4z^4 + 28(y^6z^2 + y^2z^6)\right)) \quad \text{(Eqn. S16)}$$

To test these two KH expansions for later use in this investigation (**Eqns. S15 & S16**), we applied them to the O/Fe adsorption energy at the lowest coverage of 0.25 ML and no electric field applied (0.00 V/nm)

conditions and visualized the multi-faceted Fe grain (**Figure S15**). The "bumps" in this figure correspond to a larger adsorption energy, taken directly from the DFT data.

Focusing on **Figure S15A** (3-faceted model, centered around (001) in which the first three terms in **Eqn. S15** are used), we first notice that there is symmetry on the grain, where the trend seen in the first quadrant is repeated along the entire grain surface. We also note that the three facets shown here showcase different behavior (visually, different bumps or protruding sections)—that is, their adsorption energy *magnitude* is different from one another, as expected. Adding the fourth facet changes the overall grain visual, as well (**Figure S15B**). We will denote this as the 4-faceted model in which all the terms in **Eqn. S15** are used. Adding this facet further accentuates the behavior of the grain, where the distinction between the four facets is made clearer as we increase the data included in the model. Focusing on **Figures S15C and D** now, we see that as we rotate the grain to be centered around the (011) facet, the grain visuals change again. Note that the adsorption data, itself, does not change between the (001)- and (011)-centered models. This is clear if looking at the {111} facets of **A** and **C**, for example, where the intensity of the adsorption energy for the {111} facets in the (001) oriented model look the same visually as compared to the {111} facets in the (011) oriented model since they continue to have smaller adsorption energies as compared to the {011} and the {001} facets. As such, the adsorption energies are the same regardless of the grain orientation. We do note that there is less symmetry around on the (011) oriented model than the (001) oriented model. Indeed, the (001) oriented model in 4-fold symmetric so that there are four neighboring {011} facets and four neighboring {001} facets relative to the central (001) facet. On the other hand, the (011) oriented model is 2-fold symmetric such that there are two neighboring {001} facets and two neighboring {111} facets relative to the central (011) facet.

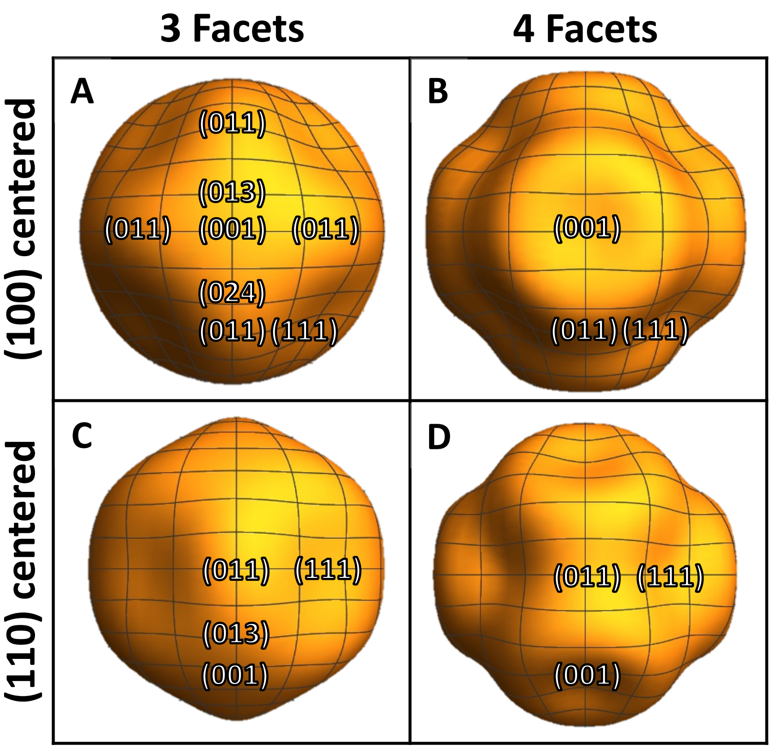

Figure S15: Kubic Harmonic visualizations of the calculated adsorption data with the inclusion of 3 facets' data (left) compared to the inclusion of 4 facets' data (right). The grain is centered at both the (001) facet (top) and (011) facet (bottom) for further comparison.

Note that the **Figure S15** KH grain models are under the following conditions: 0.25 ML and 0.00 V/nm. The grain visualizations, themselves, will change as these two parameters are altered. That is to say, not only is the grain dependent on the degree of information/data included and the rotation, but it is also dependent on the data itself, which changes as we alter both the oxygen coverage, the temperature, as well as the applied electric field. Videos of the Fe grain (centered around the (011) facet) at the four coverages as it changes with altered electric fields is included with the manuscript.

Using these KH expansions for the four tested facets gives expansion parameters based on the Miller indices. We show Fe(100) here as an example:

$$x_{100} = 1 \qquad \text{(Eqn. S17)}$$

$$y_{100} = 0 \qquad \text{(Eqn. S18)}$$

$$z_{100} = 0 \qquad \text{(Eqn. S19)}$$

$$\text{param}_1 = E_{dO}^{(0)}(1) \qquad \text{(Eqn. S20)}$$

$$\text{param}_2 = E_{dO}^{(4)}(x_{100}^4 + y_{100}^4 + z_{100}^4) = E_{dO}^{(4)} \quad \text{(Eqn. S21)}$$

$$\text{param}_3 = E_{dO}^{(6)}(x_{100}^2 y_{100}^2 z_{100}^2) = 0 \quad \text{(Eqn. S22)}$$

$$\text{param}_4 = E_{dO}^{(8)}(x_{100}^8 + y_{100}^8 + z_{100}^8) = E_{dO}^{(8)} \quad \text{(Eqn. S23)}$$

2.4 Oxygen Partial Pressure

The applied electric field is dependent on the oxygen partial pressure exposed to the grain via:

$$P_{O_2}(F) = P_{O_2}^0 e^{\beta\alpha_{O_2}(\frac{F^2}{2})} \quad \text{(Eqn. S24)}$$

where α_{O_2} is the polarizability of oxygen, found experimentally to be $0.00111\frac{\text{eVnm}^2}{\text{V}^2}$ [8]. Oxygen is a diatomic gas that interacts with the electric field via its polarizability and dipole moment, which in turn affects its partial pressure. Assuming harmonic vibrations, for a single molecule, the Hamiltonian is given as:

$$h = \frac{\overrightarrow{P^2}}{2M} + \frac{\overrightarrow{p^2}}{2\mu} + \varepsilon_e + \frac{k}{2}(r - r_e)^2 - \vec{F}\cdot\vec{d} - \frac{1}{2}\vec{F}\cdot\overleftrightarrow{\alpha}\cdot\vec{F} \quad \text{(Eqn. S25)}$$

where $\vec{P}$ is the linear momentum of the molecule's center of mass, M is the mass, $\vec{p}$ is the relative momentum, μ is the relative mass, $\vec{F}$ is the electric field, $\vec{d}$ is the dipole moment, and $\overleftrightarrow{\alpha}$ is the polarizability. Arrows indicate a vector. We can define $\overleftrightarrow{\alpha}$ as a matrix, where the z-axis joins the nuclei of both oxygen molecules:

$$\overleftrightarrow{\alpha} = \begin{pmatrix} \alpha_\perp & 0 & 0 \\ 0 & \alpha_\perp & 0 \\ 0 & 0 & \alpha_\parallel \end{pmatrix} \quad \text{(Eqn. S26)}$$

where $\alpha_\perp$ and $\alpha_\parallel$ are the polarizabilities perpendicular and parallel to the interatomic axis, respectively. This allows us to define the electric field as:

$$\vec{F} = F\,(-\cos\phi\sin\theta, -\sin\phi\sin\theta, \cos\theta) \quad \text{(Eqn. S27)}$$

Now we can convert the Hamiltonian into spherical coordinates:

$$h = \frac{\overrightarrow{P^2}}{2M} + \frac{p_r^2}{2\mu} + \varepsilon_e + \frac{k(r-r_e)^2}{2} + \frac{(p_\theta^2 + \frac{p_\phi^2}{\sin^2\theta})}{2I} - Fd\cos\theta - \frac{F^2}{2}[\alpha_\perp + (\alpha_\parallel - \alpha_\perp)\cos^2\theta] \quad \text{(Eqn. S28)}$$

where the moment of inertia $I = \mu r_e^2$, θ is the polar angle with respect to the z-axis, and ϕ represents the azimuth angle with respect to the z-axis. This allows us to separate the Hamiltonian into its translational

($\frac{\overrightarrow{P^2}}{2M}$), vibrational ($\frac{p_r^2}{2\mu} + \varepsilon_e + \frac{k(r-r_e)^2}{2}$), and rotational ($\frac{(p_\theta^2 + \frac{p_\phi^2}{\sin^2\theta})}{2I} - \mathrm{Fdcos}\theta - \frac{F^2}{2}[\alpha_\perp + (\alpha_\parallel - \alpha_\perp)\cos^2\theta]$) components.

In the canonical ensemble at $\beta = \frac{1}{k_B T}$, the partition function of a gas that contains N molecules in a space of volume V with an electric field $\vec{F}$ is given by:

$$Z = \frac{1}{N!}(\zeta_{trans}\zeta_{vib}\zeta_{rot}\zeta_{elect}\zeta_{nucl})^N \quad \text{(Eqn. S29)}$$

The one-molecule translation, vibration, and rotation partition functions are given by:

$$\zeta_{trans} = V\frac{(2\pi M k_B T)^{\frac{3}{2}}}{h^3} \quad \text{(Eqn. S30)}$$

$$\zeta_{vib} = \frac{e^{-\beta\varepsilon_e}}{2\sinh\frac{\beta\hbar\omega}{2}} \quad \text{(Eqn. S31)}$$

$$\zeta_{rot} = \frac{1}{sh^2}\int e^{-\beta h_{rot}} dp_\theta d\theta dp_\phi d\phi \quad \text{(Eqn. S32)}$$

where $\hbar = \frac{h}{2\pi}$ is Planck's constant, $\omega = \sqrt{\frac{k}{\mu}}$ is the angular frequency of vibrations, and $s = 2$ for oxygen (a homonuclear molecule). With $s = 2$, we get:

$$\zeta_{rot} = \frac{Ik_B T}{s\hbar^2}e^{\frac{\beta F^2\alpha_\perp}{2}}\int_{-1}^{+1} e^{\beta F d\lambda} e^{\frac{\beta F^2(\alpha_\parallel - \alpha_\perp)\lambda^2}{2}} d\lambda \quad \text{(Eqn. S33)}$$

when $F = 0$, $\zeta_{rot} = \frac{Ik_B T}{s\hbar^2}$, allowing us to define an effective potential:

$$u_{eff} = -\frac{\alpha_\perp}{2}F^2 - \frac{1}{\beta}\ln\int_0^1 \cosh(\beta F d\lambda) e^{\frac{\beta F^2(\alpha_\parallel - \alpha_\perp)\lambda^2}{2}} d\lambda \quad \text{(Eqn. S34)}$$

At low electric fields:

$$u_{eff} = -\left(\frac{\alpha_\perp}{3} + \frac{\alpha_\parallel}{6} + \frac{\beta d^2}{6}\right)F^2 + \mathcal{O}(F^4) \quad \text{(Eqn. S35)}$$

and at high fields:

$$u_{eff} \cong -\frac{\alpha_\parallel}{2}F^2 \text{ if } \alpha_\parallel > \alpha_\perp \quad \text{(Eqn. S36)}$$

$$u_{eff} \cong -\frac{\alpha_\perp}{2}F^2 \text{ if } \alpha_\parallel < \alpha_\perp \quad \text{(Eqn. S37)}$$

Assuming oxygen behaves as an ideal gas ($P = nk_B T$) and the free energy, $F = -k_B T \ln Z$, the density of molecules is given by:

$$n = \frac{N}{V} = \frac{g}{h^3}(2\pi M k_B T)^{\frac{3}{2}} \zeta_{vib} \zeta_{rot} e^{\beta\mu} \quad \text{(Eqn. S38)}$$

where $g = \zeta_{elect}\zeta_{nucl}$. Because ζ_{rot} depends on position due to the electric field, the pressure and density must also depend on position:

$$P(\vec{r}) = P(\infty)e^{-\beta u_{eff}(\vec{r})} = n(\vec{r})k_B T \quad \text{(Eqn. S39)}$$

where $P(\infty)$ is the pressure at an arbitrarily large distance where the electric field vanishes and the u_{eff} is given by **Eqn. S34** and is approximated at high fields for this investigation.

2.5 Reaction Kinetics

2.5.1 Equilibrium Model

The reaction modeled for this investigation is the adsorption of oxygen onto Fe:

$$O_2(g) + 2\emptyset(ad) \rightleftharpoons 2O(ad) \quad \text{(Eqn. S40)}$$

where $\emptyset$ indicates an adsorption site. To model the kinetics of this reaction, the following differential equation is utilized:

$$\frac{d\theta_O}{dt} = 2k_{aO_2}P_{O_2}(1-\theta_O)^2 - 2k_{dO_2}^0\theta_O^2 \quad \text{(Eqn. S41)}$$

assuming no precursor state of oxygen. Here k_{aO_2} and k_{dO_2} are the adsorption and desorption rate constants, respectively, defined as:

$$k_{aO_2} = k_{aO_2}^0 e^{-\beta E_a^\ddagger} \quad \text{(Eqn. S42)}$$

$$k_{dO_2} = k_{dO_2}^0 e^{-\beta E_d^\ddagger} = k_{dO_2}^0 e^{-\beta(\widetilde{E_d}+E_a^\ddagger)} \quad \text{(Eqn. S43)}$$

where $\beta = \frac{1}{RT}$, $E_d^\ddagger$ is the total energy for oxygen desorption on the Fe grain surface, $\widetilde{E_d}$ is the binding energy of two oxygen atoms, and $E_a^\ddagger$ is the activation energy for oxygen dissociation. Here, $\widetilde{E_d} = -2E_{ads,O}$, taken from the DFT calculations.

For the equilibrium case, we set $\frac{d\theta_O}{dt} = 0$, giving the new oxygen coverage equation (first by plugging in those three definitions):

$$\theta_{O,equil} = \frac{1}{1 + \sqrt{\frac{k_{dO_2}^0 e^{-\beta\widetilde{E_d}}}{k_{aO_2}^0 P_{O_2}}}} \quad \text{(Eqn. S44)}$$

The adsorption and desorption rate pre-exponential factors, $k_{aO_2}^0$ and $k_{dO_2}^0$, are defined as:

$$k_{aO_2}^0 = \frac{S_O^0 a_s}{\sqrt{2\pi m_{O_2} k_B T}} \quad \text{(Eqn. S45)}$$

$$k_{dO_2}^0 = \frac{S_O^0 a_s k_B T Z_{int}}{\lambda^2 h q_{O,ads}^2} \quad \text{(Eqn. S46)}$$

where S_O^0 is the initial sticking coefficient, estimated at 0.05, based on Pt(111), T is the temperature, k_B is the Boltzmann constant, m_{O_2} is the mass of molecular oxygen, and h is Planck's constant. The thermal wavelength, λ, is calculated via:

$$\lambda = \frac{h}{\sqrt{2\pi m_{O_2} k_B T}} \quad \text{(Eqn. S47)}$$

Z_{int} is the partition function for oxygen in the gas-phase, shown in **Eqn. S48**, and $q_{O,ads}$ is the partition function for the center-of-mass vibrations of adsorbed oxygen, shown in **Eqn. S49**:

$$Z_{int} = q_{rot}^{O_2} \cdot q_{vib}^{O_2} \quad \text{(Eqn. S48)}$$

$$q_{O,ads} = (q_{vib}^{O_{ads}})^{\frac{2}{N_O}} \quad \text{(Eqn. S49)}$$

where $q_{rot}^{O_2}$ (**Eqn. S50**) and $q_{vib}^{O_2}$ are the rotational and vibrational partition functions for gas-phase molecular oxygen, and $q_{vib}^{O_{ads}}$ is the vibrational partition function for atomic adsorbed oxygen; N_O is the number of adsorbed oxygen atoms.

$$q_{rot}^{O_2} = \frac{8\pi^2 I_{O_2} k_B T}{h^2} \quad \text{(Eqn. S50)}$$

$$q_{vib}^{j} = \prod_i \frac{e^{-\frac{h\nu_i^j}{2k_BT}}}{1 - e^{-\frac{h\nu_i^j}{k_BT}}} \quad \text{(Eqn. S51)}$$

where I_{O_2} is the moment of inertia for molecular oxygen; ν_i^j are the vibrational frequencies, calculated via DFT; and j can represent either molecular or adsorbed oxygen to account for both $q_{vib}^{O_2}$ and $q_{vib}^{O_{ads}}$. From these sets of equations, the oxygen coverage was modeled under both equilibrium and non-equilibrium conditions.

2.5.2 Precursor-Mediated Model

2.5.2.1 Notes on the 4-faceted model used in Figures 3 and 5.

Blonski et al.[74] found the dissociative adsorption of oxygen on Fe(100) and Fe(110) to be either barrierless over a full monolayer of coverage (for Fe(100)) or to have a small barrier (for Fe(110)) at similar coverages shown here. The difference in adsorption energy at 0 V/nm for Fe(111), compared to Fe(100), is

negligible (except for the case of 0.25 ML where the adsorption strength is weaker by 0.27 eV). Hence, we assume barrierless adsorption in this study for all facets. Therefore, the time evolution of O on the grain was simulated using differential **Eqn. S41**. On the opposite trend, we observe that the Fe{024} facets and the Fe{013} facets remain unscathed by the oxidation which confirms our first principles-based model predicting a larger increase of the adsorption energy over the Fe{024} than the other facets tested (**Fig. 2B**) when an external electric field is applied.

2.5.2.2 Adsorption Kinetics and Sticking Coefficient Modeling

In the dissociative adsorption model that is presented in **Figure 3** of the main manuscript, we are assuming that the coverage dependence of the sticking coefficient, $S(\theta_O)$, is:

$$\frac{S}{S_o} = (1 - \theta_O)^2 \quad \text{(Eqn. S52)}$$

where S_o is the initial coefficient[75]. We have further compared the sticking coefficient to the experimental results of the sticking coefficient and a precursor-mediated model to determine whether such an assumption for the initial sticking coefficient is valid. In such a model, the precursor intermediate is taken into account through O_2(pre):

$$O_2(\text{gas}) + \text{surface} \underset{\tilde{k}_d}{\overset{\tilde{k}_a}{\rightleftharpoons}} O_2(\text{pre}) + \text{surface} \quad \text{(Eqn. S53)}$$

$$O_2(\text{pre}) + 2\emptyset(\text{ad}) \underset{k_d}{\overset{k_a}{\rightleftharpoons}} 2O(\text{ad}) \quad \text{(Eqn. S54)}$$

Inclusion of the precursor states requires modification of the differential **Eqn. S41**. In this model, we assume that $\frac{d\theta_{O_2}}{dt} = 0$. With this assumption, we obtain the following coverage dependence of the precursor state:

$$\theta_{O_2} = \frac{\tilde{k}_a P_{O_2} + k_d {\theta_o}^2}{\tilde{k}_d + k_a {\theta_\emptyset}^2} \quad \text{(Eqn. S55)}$$

The time evolution of the oxygen coverage will then be given by:

$$\frac{d\theta_o}{dt} = \frac{2}{1 + K{\theta_\emptyset}^2}\left(\tilde{k}_a K P_{O_2} {\theta_\emptyset}^2 - \tilde{k}_d k_d {\theta_o}^2\right) \quad \text{(Eqn. S56)}$$

where *K* is given by J.-S. McEwen et al.[8,76]:

$$K = \frac{k_a}{\tilde{k}_d} = k_o e^{-\beta(E_k + A_o \theta_o)} \quad \text{(Eqn. S57)}$$

From this, we can obtain the sticking coefficient:

$$\frac{S}{S_o} = \frac{\frac{K}{k_o e^{-\beta E_k}}(1-\theta_o)^2(1+k_o e^{-\beta E_k})}{1+K(1-\theta_o)^2} \quad \text{(Eqn. S58)}$$

where the initial sticking coefficient is given by:

$$S_o = \frac{k_o e^{-\beta E_k}}{(1+k_o e^{-\beta E_k})} \quad \text{(Eqn. S59)}$$

We have adjusted the values of E_k, A_o and k_o to -0.042 eV, 0.45 eV/ML and 1.0 to get the best fit to the experimental data. A comparison of this model to the experimental coverage-dependent sticking data is given in **Figure S16**. We find that the relation given in **Eqn. S58** accurately reproduces the experimental sticking coefficient for coverages below 0.15 ML but underestimates it above 0.15 ML, moreover, it approaches zero above 0.40 ML. If one models the sticking coefficient using the model given in **Eqn. S52**, we find that the sticking coefficient decreases with increasing coverage in a similar manner as compared to experiment, although the sticking coefficient reaches zero at 1.0 ML unlike in the experiment where it attains a constant value of about 0.3 at 0.40 ML and above. Additionally, when one models the sticking coefficient with the dissociative adsorption model given in **Eqn. S52**, we find that this model overestimates the sticking coefficient up to about 0.60 ML and underestimates it above 0.60 ML. The discrepancy for coverages above 0.60 ML could be due the formation of the oxide layer, which is not considered in either model. Indeed, taking the formation of the oxide into account would increase the sticking coefficient value. As such, neither model adequately captures the adsorption kinetics for coverages beyond 0.60 ML as one would need to account for the formation of an oxide. As for the discrepancy at coverages below 0.6 ML when modeling the coverage dependence by **Eqn. S52**, it is likely due to the absence of lateral interactions in in the adsorption kinetics. We note that when one sets A_o to be 0.45 eV/ML (which corresponds to repulsive lateral interactions), one gets the correct coverage dependence for the sticking coefficient for coverages below 0.1 ML when using **Eqn. S58**. However, such a value of A_o does not adequately model the coverage dependence for coverages beyond 0.1 ML since the sticking rapidly goes to 0 while the experimental data does not. As such, the lateral interactions are not adequately described when using the model given by **Eqn. S58** as well since they are too repulsive. Thus, neither model quantitatively captures the experimental data for coverages below 0.6 ML.

Interestingly, we have also found that the coverage dependence of the sticking coefficient is qualitatively similar to that given by **Eq. S52** if one sets $A_o = 0$ in **Eq. S58** for two sets of parameter values: (1) k_o = 1 and E_k values ranging from -0.02 eV or 5 eV, (2) k_o = 0.1 and E_k values ranging from -0.05 eV to 5 eV. Further, at k_o = 0.1 and E_k = 0, the sticking coefficient coverage dependence is virtually equal to that of **Eq. S52** at all coverage values. As such, the values of E_k, A_o, and k_o would need to be precisely

determined by other means for the precursor-mediated model state since there are a range of values that would give a similar coverage dependence to what is given in **Eq. S52**. Thus, we have opted to model the adsorption kinetics using the model that is given by **Eq. S52** since it does not involve any unknown parameters and it also qualitatively captures the coverage dependence of the sticking coefficient as compared to the experimental results for coverages up to 0.60 ML.

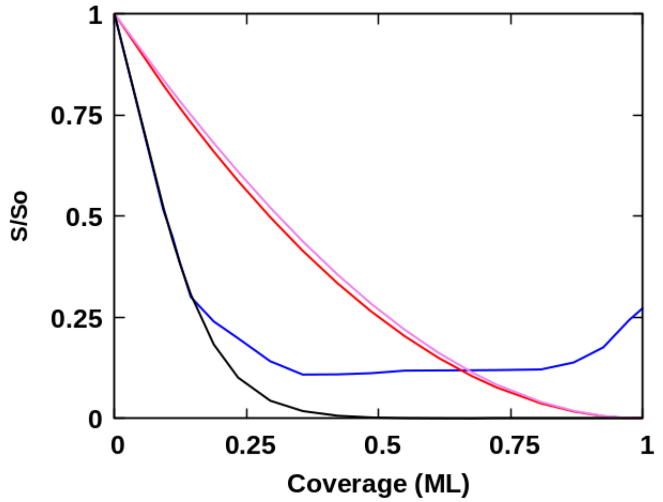

Figure S16: Ratios of the coverage dependent sticking coefficient to the initial sticking coefficient. (1) Blue, (2) black, (3) red, and (4) violet lines are: (1) the experimentally determined values[77], (2) that obtained from **Eq. S58** (E_k, A_o and k_o values of -0.042 eV, 0.45 eV/ML and 1.0, respectively), (3) that obtained from **Eq. S52** and (4) that obtained from **Eq. S58** in which K has no temperature and no coverage dependence (where E_k, A_o and k_o are set to values of 0, 0 and 0.10, respectively).

2.6 Normalized Surface Plots of Oxygen Coverage

2.6.1 Determining the Adsorption/Desorption Equations

To model the oxygen coverage on the multi-faceted grain using the combined DFT data and experimental conditions, we must first determine how to incorporate the dependence on both the oxygen coverage as well as the applied electric field. The following equation was used:

$$\mathrm{E_{ads}} = \mathrm{A}(\theta - 0.25) + \mathrm{B}(\theta - 0.25)^2 + \mathrm{CF} + \mathrm{DF}^2 + \mathrm{E}(\theta - 0.25)\mathrm{F} + \mathrm{E_{ads}}(0.25\ \mathrm{ML}, 0\ \mathrm{V/nm}) \quad \text{(Eqn. S60)}$$

where each coefficient ($\mathrm{A, B, C, D, E, E_{ads}}(0.25\ \mathrm{ML}, 0\ \mathrm{V/nm})$) is a representation of the KH coefficient for the expansion. For example, to determine the coefficient A for the (100) facet, the following equation is used:

$$A(Fe(100)) = A_1(1) + A_2\left(x^4 + \frac{1}{2}y^4 + 3y^2z^2 + \frac{1}{2}z^4\right) + A_3\left(x^2\frac{(y^2 - z^2)^2}{4}\right) + A_4(x^8 + \frac{1}{8}(y^8 + z^8 + 70y^4z^4 + 28(y^6z^2 + y^2z^6)) \quad \text{(Eqn. S61)}$$

Here, the constants $A_1, A_2, A_3,$ and A_4 are used, along with the Miller indices, (hkl), taken to represent (x, y, z), to determine the overall coefficient, A for the Fe(100) facet. This is repeated for all coefficients; the coefficients are fit to **Eqn. S16** and set to the data for that facet to find all the coefficients. This is repeated for all facets. The coefficients, their Test Square Error (TSE) as well as their Leave-One-Out Cross Validation (LOO-CV) score and RMSE are shown in **Table S2**. The RMSE indicates how well the data fits the model, where a lower RMSE value indicates a better model fit. A LOO-CV score, on the other hand, determines how predictable a given model is to data not included in the training set; here the LOO-CV scores are relatively low and thus the model is likely to be highly predictable—an important consideration since the coverage models extrapolate to the entire Fe grain. These coefficients were found to be suitable for the investigation and were thus used to determine the desorption rates of oxygen on each facet.

Although the facets have been shown to behave differently from one another, this investigation looks to model an entire Fe grain (which is why KH were used to expand the data from these chosen facets to the entire Fe grain by taking advantage of the symmetry of the bcc lattice). As such, instead of modeling the desorption of oxygen separately for each facet and thus doing separate KH expansions for each facet coefficient, the expansion for each coefficient ($A, B, C, D, E, E_{ads}(0.25\ ML, 0\ V/nm)$) was done for all facets at once, where the combined expansion was then used in one desorption equation (**Eqn. S60**) to model over the entire Fe grain.

2.6.2 Normalizing Oxygen Coverage Across All Facets

Table S3: Coefficients for the oxygen desorption equations for each facet, along with the error analyses.

	**Fe(100)**	**Fe(110)**	**Fe(111)**	**Fe(024)**
**A**	0.330	1.940	-0.139	1.154
**B**	0.200	-0.435	0.122	-0.990
**C**	0.033	0.0750	0.005	0.122
**D**	0.097	0.104	0.103	0.139
**E**	-0.0195	0.001	0.018	-0.001
$\mathbf{E_{ads}(0.25ML, 0\frac{V}{nm})\ [\frac{eV}{\theta}]}$	-2.531	-2.601	-2.606	-2.785
$\mathbf{TSE\ [\frac{eV}{\theta}]^2}$	0.001	0.001	0.001	0.009

**RMSE** $[\frac{eV}{\theta}]$	0.033	0.0086	0.023	0.079
**LOO-CV** $[\frac{eV}{\theta}]$	0.037	0.010	0.026	0.090

The DFT data and the KH models were combined, along with experimental conditions, to simulate the distribution of oxygen on the entire multi-faceted catalytic Fe grain under both equilibrium and non-equilibrium conditions. Before this could be done, there were certain conditions that needed to be undertaken. First, since the Fe facets, themselves, have different surface areas, the surface coverage needs to be normalized across all facets. This was done via:

$$\theta = \Theta \frac{a_s}{A(hkl)} \qquad \text{(Eqn. S62)}$$

where θ is the normalized oxygen coverage used in all kinetic equations, Θ is the previously defined coverage that does not take facet area into account, A(hkl) is the area of each respective facet, calculated according to **Eqn. S63**, and a_s is the smallest surface area, taken as the surface area for Fe(110), that normalizes the oxygen coverage.

$$A(hkl) = \frac{a^2}{4}[3 + (-1)^{h+k+l}]\sqrt{h^2 + k^2 + l^2} \qquad \text{(Eqn. S63)}$$

where a is the lattice constant for Fe and h, k, l are the Miller indices. Using this equation, the surface areas for Fe(100), Fe(110), Fe(111), and Fe(024) are: 8.23 $Å^2$, 5.82 $Å^2$, 14.25 $Å^2$, and 18.37 $Å^2$, respectively.

2.6.3 Normalized Surface Oxygen Distribution

Figure S17 shows the time evolution of the oxidation of a field emitter tip. This figure is similar to Figure 3 (both with 40 contour lines), but with one key difference: the contour lines in Figure S17 were generated uniformly between coverage values of 0 and 1 ML, whereas those in Figure 3 were automatically generated by the plotting program. We present both results because the contour lines in Figure 3 help compare with experimental data, while those in Figure S17 illustrate that the coverage differences between contour lines near the apex of the tip in Figure 3 are minimal. This is because the system is already close to equilibrium after 1 second, as shown in the time evolution plot in Figure 5 at the corresponding electric field value.

We also note that in all other plots in the manuscript and supporting information, contour lines were automatically generated by the plotting program using 12 contour lines.

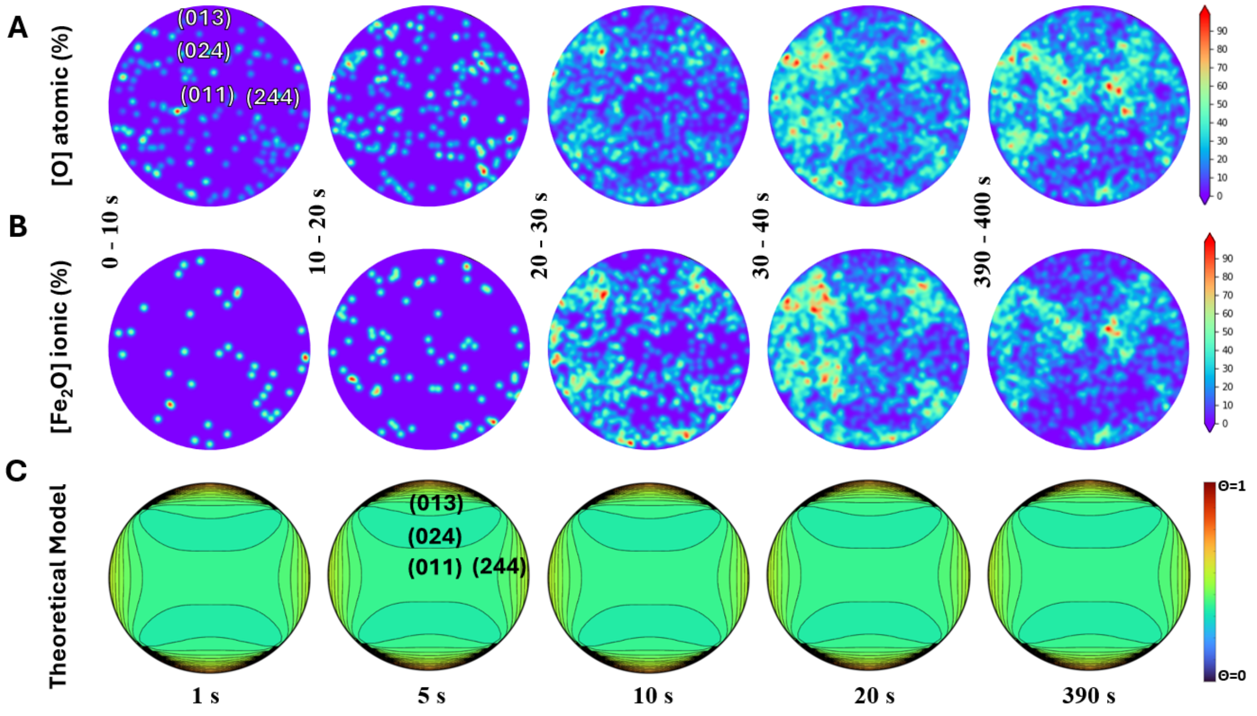

Figure S17: Experimentally measured 2D projection composition maps of (A) O(%)atomic, (B) Fe_2O^{n+} ionic of a Fe specimen exposed to 1.2×10^{-7} mbar of pure O_2 with a constant ~18 V/nm (~23.4 V/nm pulsed) applied EF estimation at 303K. The experimental composition maps shown are displaying the average composition detected over a 5 min time-lapse (C) Theoretically derived 2D projection composition maps from corresponding first principles-based model of evolving oxygen coverage over a hemispherical field emitter tip with an EF of 24.12 V/nm, a pressure of 1.1×10^{-8} mbar and a temperature of 300 K. We note that the displayed contour lines were generated at 40 fixed contour line values evenly spaced between 0 and 1. The corresponding plot with automatically generated contour lines is shown in Figure 3.

2.6.3.1 The 4-Facet Model at 1.1×10^{-9} mbar with No Adjustment of the O Binding Energies Using the Paraboloidal Model of the Field Emitter Tip

The oxygen distribution over the surface of the multi-faceted catalytic grain was modeled under both equilibrium and non-equilibrium conditions for comparison to experimental efforts. In this subsection, we demonstrate the consequences of not uniformly reducing the adsorption energies by 1.2 eV, as was done in the main text. Here we include the models at an oxygen partial pressure of 1.1×10^{-9} mbar. In addition to decreasing the pressure, one needs to increase the electric field strength to 30.5 V/nm as well as the temperature to 700 K so that the coverage distribution plots that are similar to the experimental results. Time evolution simulations generally take much longer to reach equilibrium in comparison to the simulations at 1.1×10^{-8} mbar where the oxygen binding energies are uniformly reduced (**Figure 5**). We

also note that we assumed that the shape of the tip is a paraboloid in **Figure S18**. If one uses a hemispherical model, we do note that there are some differences in the coverage distribution (not shown) although the needed temperature, pressure and electric field values are similar in order to get oxygen coverages distribution plots that are similar to what is seen experimentally. We also show a more detailed comparison between the time evolution at 25 V/nm and 30.5 V/nm in **Figure S19**. Finally, in **Figure S20**, we show the effect of increasing the oxygen pressure on the time evolution dynamics, which also allows us to reduce the electric field strength to get a pattern that is like what is seen experimentally.

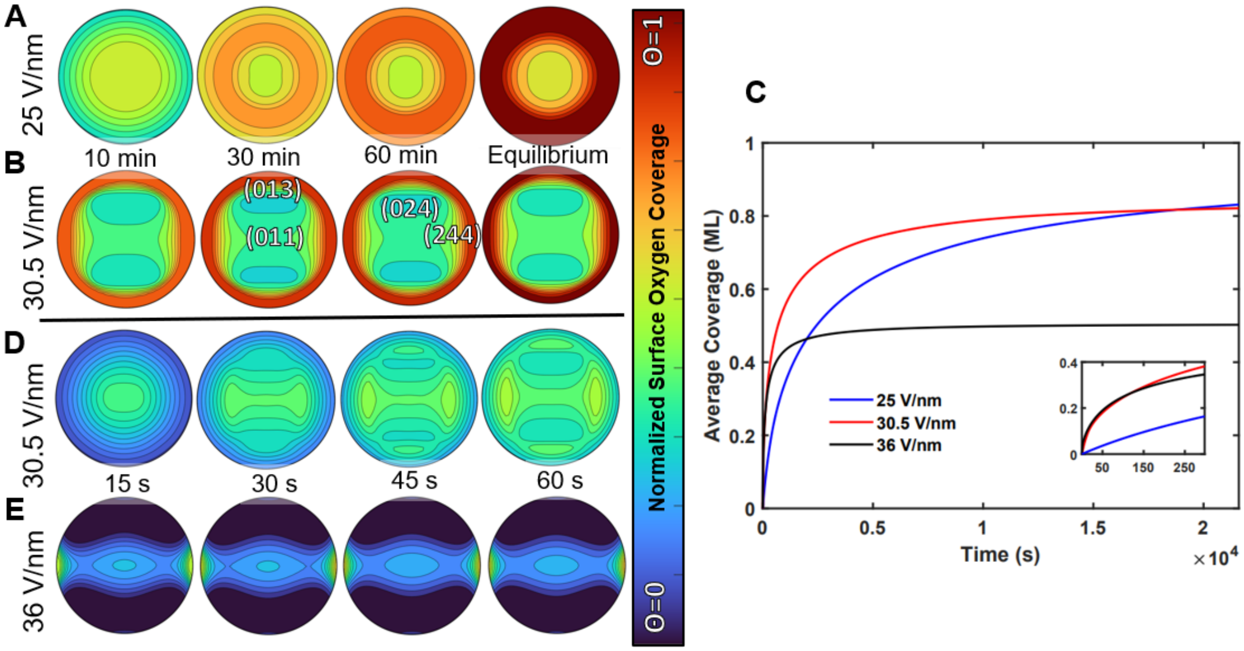

Figure S18: Snapshots of the time evolution of oxygen coverage on a paraboloidal Fe grain at 1.1×10^{-9} mbar and $700\ \mathrm{K}$ under a **A** $25\ \mathrm{V/nm}$ and **B** $30.5\ \mathrm{V/nm}$ applied electric field in which the binding energies of O are taken from the DFT-based values on metallic surfaces. **C** Time evolution of oxygen coverage under different electric fields. Snapshots of time evolution of oxygen coverage on the multifaceted grain for the first 60 seconds for the **D** 30.5 V/nm-scenario and **E** 36 V/nm-scenario. We note that the displayed contour lines were generated automatically within the plotting software.

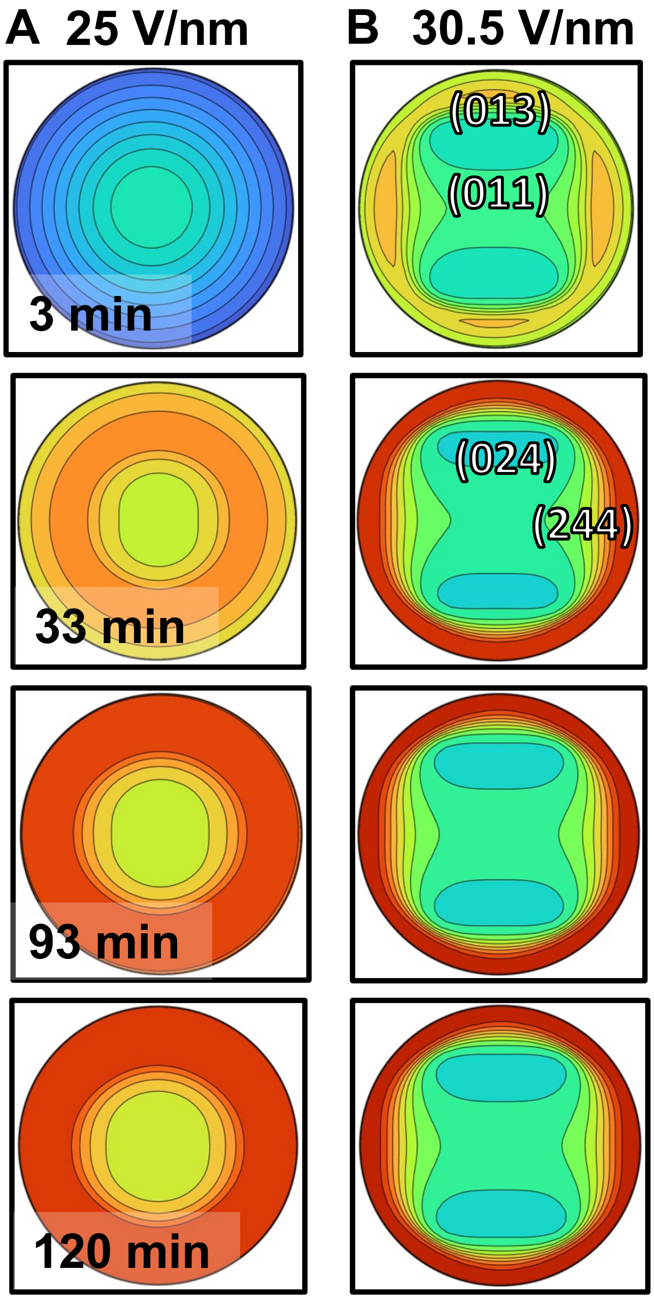

Figure S19: Snapshots of the normalized surface oxygen distribution on a paraboloidal Fe grain in which the O binding energies are taken from the DFT-based values on metallic surfaces. The oxygen pressure was fixed at 1.1×10^{-9} mbar and the temperature at 700 K for the 4-facet models under **A.** a 25 V/nm applied field, and **B.** a 30.5 V/nm applied field. We note that the displayed contour lines were generated automatically within the plotting software.

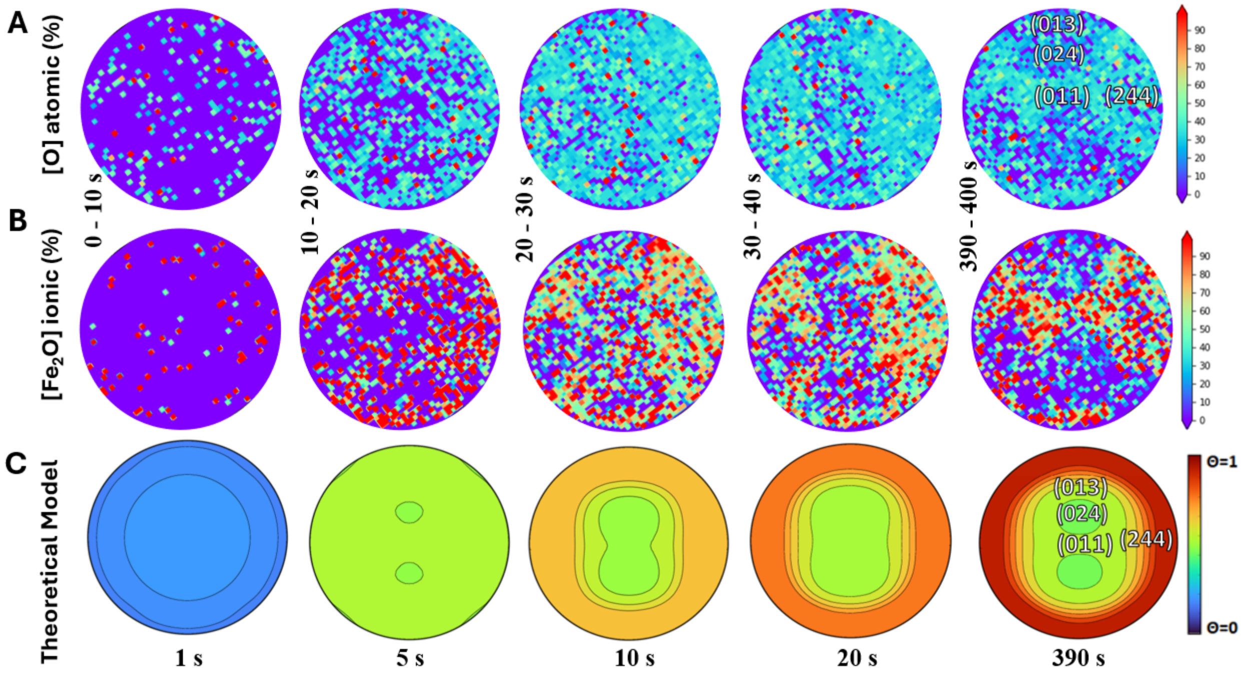

Fig. S20: Experimentally measured 2D projection composition maps of (A) O(%)atomic, (B) Fe_2O^{n+}ionic of a Fe specimen exposed to 1.2×10^{-7} mbar of pure O_2 with a constant 15 V/nm (21.8 V/nm pulsed) applied EF at room temperature. The experimental composition maps shown are displaying the average composition detected over a 5 min time lapse (C) Theoretically derived 2D projection composition maps from corresponding first principles-based model of evolving oxygen coverage over a field emitter tip with an EF of 28 V/nm, a pressure of 1.1×10^{-8} mbar and a temperature of 700 K over a paraboloidal model of a field emitter tip. We note that the displayed contour lines were generated automatically within the plotting software.

2.6.3.2 Comparing the 4-Facet & 3-Facet Equilibrium Models on a Hemispherical Model of a Field Emitter Tip

In the '3-facet' model, the KH were only expanded to include data from three facets (that is, Fe(100), Fe(110), and Fe(111)), instead of the four facets which we have used in the main text (which includes the three previously mentioned facets and Fe(024)). However, KH[43] allow us to extrapolate to other facets on the grain of which there is no data for using the symmetry of the Fe grain.

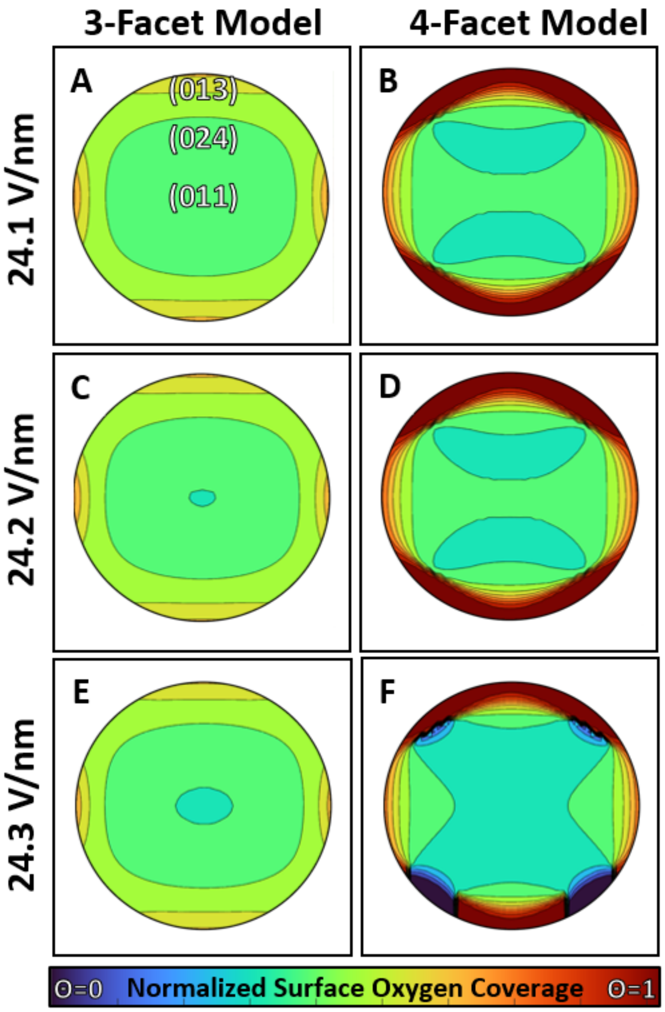

Figure S21: Normalized surface oxygen distribution on a hemispherical model of an Fe grain at equilibrium at 1.1×10^{-8} mbar and 300 K for a 3-facet model under **A** 24.1 V/nm field, **C** 24.2 V/nm field, and a **E** 24.3 V/nm field, and a 4-facet model under **B** 24.1 V/nm field, **D** 24.2 V/nm field, and a **F** 24.3 V/nm field. We note that the displayed contour lines were generated automatically within the plotting software.

We simulated the equilibrium conditions using a '4-facet' model and compared it to the '3-facet' model at 1.0×10^{-8} mbar and 300 K for three applied field values, 24.1 V/nm, 24.2 V/nm, and 24.3 V/nm (**Figure S21**). At the lower applied electric field of 20 V/nm, the differences between the 3-facet and 4-facet models (**Figure S21 A** and **B**, respectively) are distinct. Although the pattern of oxygen coverage is similar to one another, the 3-facet model shows an overall lower oxygen coverage compared to the 4-facet model (i.e., more green coloring). Importantly, the trends seen for Fe(013) and Fe(024) are different between the two models—where the 3-facet model shows less oxygen coverage for Fe(013) as compared to the 4-facet model and slightly more coverage for Fe(024) in the 3-facet model as compared to the 4-facet model--but

for Fe(011) the differences are negligible. This difference in oxygen coverage continues as we increase the EF to 24.2 V/nm (**Figure S21 C** and **D**) and even to 24.3 V/nm (**Figure S21 E** and **F**), where the trends remain consistent. Overall, the two models show consistent patterns on the Fe grain, but the magnitude of oxygen coverage is not consistent between the two, except for at the central facet.

Compared to the experimental results (**Figure 3A** at 90-95 minutes), the oxygen coverage trends for the 4-facet model (**Figure S21D**) are more consistent than those for the 3-facet model (**Figure S21C**). This is most notable at the (011) facet where both the experimental and 4-facet model show that the central facet on the Fe grain, (011) has a higher oxygen coverage than the facets north and south of it (i.e., Fe(013) and Fe(024))), whereas the 3-facet model shows the opposite trend. There are two potential causes for this deviation in trends regarding the addition of the fourth facet—Fe(024)—to the model. Firstly, the $E_{ads}(\theta)$ seen for Fe(024) shows that Fe(024) does not see the same general trends in adsorption energy (**Figure 2A**) as the other facets (i.e., its largest adsorption energy does not occur at exactly 0 V/nm), so its oxygen coverage behavior is not likely to behave similarly to the other facets—potentially a result of step facets behaving differently in adsorption behavior compared to 'flatter' facets, like (011) and (111). The DFT data for Fe(024) (**Figure 2B**) also shows that Fe(024) is most impacted by the application of positive electric fields. In these surface plots (**Figure S21 B, D, F**), we see that the coverage behavior changes most for Fe(024) compared to the other facets. So, it is possible that its inclusion in this model caused the entire grain to become more sensitive to applied fields. The other likely scenario is an issue regarding the applicability of the KH. In previous work mapping the oxygen coverage as it depends on applied electric fields[20] only low-Miller index facets ({001}, {011}, and {111}) were used for the model. von der Lage and Bethe[43], who developed the KH approach, tested this model on sodium at a low Miller index. Our results indicate that in order to have accurate results on the stepped facets, one needs to also include stepped facets in the expansion. Thus, the expansion is quite sensitive to the number of terms that is included in it and needs to be guided by the experimental results in order to be predictive. For comparison purposes, we also compare the time evolution of the oxidation of the Fe field emitter tip at other field values in **Figure S19** and **Figure S20**.

3. Supporting information for the method section

3.1 Principles of Operando Atom Probe

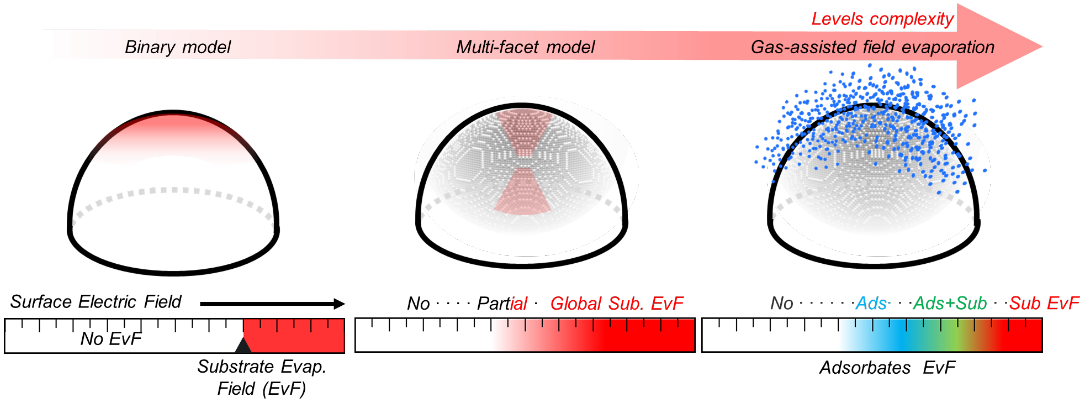

Figure S22: Schematic representation of field evaporation complexity. In the binary model we consider only the electric field distribution along the global specimen geometry. In the multi-facet model, we take into account the specific local structure, i.e. different crystallographic facets. Finally, we need to consider the presence of reactive gas that will chemically interact with the surface and ease the field evaporation.

3.2 Operando Atom Probe experimental workflow

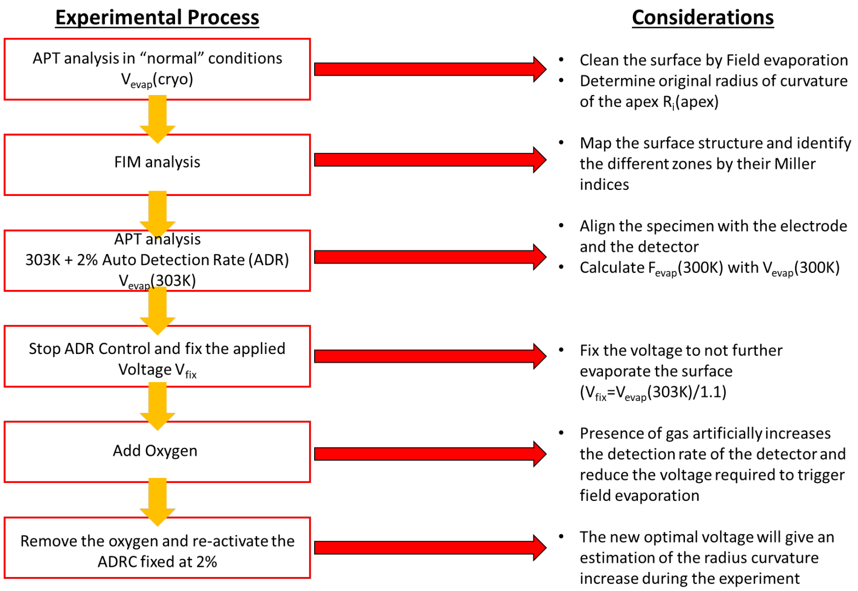

Figure S23: Operando Atom Probe workflow schematic.

Description of the experimental workflow.

The experimental workflow is shown in Figure S23. After fabrication, the Fe specimen is at first ran in the LEAP4000 in voltage mode at 45 K, with 10% pulse amplitude and 125kHz pulse frequency and 0.2% detection rate. The objective of this first step is to clean the specimen and verify that the surface is clean (low presence of water and other contaminants) and that the surface is "monocrystalline" e.g. without surface defect like small dislocations. This is observable in APT with the presence of lines of symmetry in

the event density map. Finally, we need to be sure that the voltage at which we can exploit the specimen is sufficiently low to last during the different treatment phases of the experiment. Typically, Fe specimen without a voltage of evaporation higher than 5kV are discarded. The total voltage (V_{stat} + V_{pulse}) used to provoke the field evaporation of the Fe specimen is used as benchmark to estimate the electric field afterward. We rely on the electric field found in literature to establish the benchmark. The second step consists of the FIM imaging of the specimen at 45 K using Ne. Third step, we move the specimen away from the counter electrode, power-off the cryo-compressor and stabilize the stage temperature at 300 K which set the specimen at 303 K according to the aperture's sensor. We perform a regular APT analysis of the specimen. Because of the absence of cold surfaces in the chamber under these conditions, the vacuum of the chamber increases from $2.6<10^{-11}$ mbar to $\sim 5\times 10^{-11}$ mbar. Then we decrease the voltage 9% with a maximum of 500V. With this voltage difference, we estimate that the surface is safe from field evaporation. Then we introduce the O_2 in the chamber at the target pressure. At this point the detection rate increases naturally, up to 0.2% even if we do not get any peak or signal coming from the surface. Then the voltage is slowly increased until we start to observe discernable peaks. Regardless of the intensity of the background noise, the presence of peaks is logically linked to a phenomenon happening at the surface on which we apply the voltage pulses. This voltage is noted, and the APT run stopped. This is this final voltage that is then used as a constant voltage for an EAP experiment.

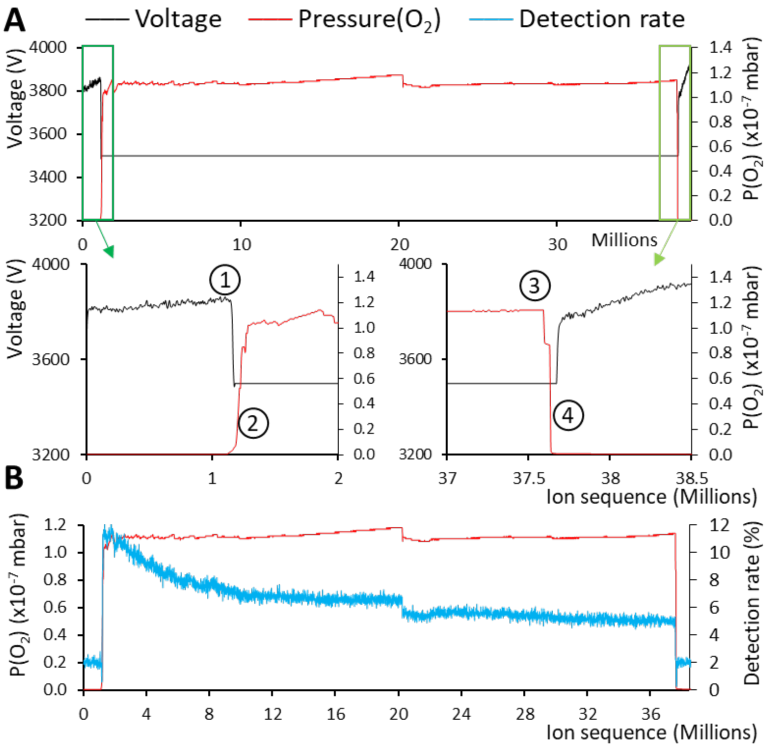

Figure S24: A. Voltage and Pressure plots of the EAP experiment presented in Fig.3. (1) After a regular field evaporation cleaning with a targeted detection rate of 2% (setting the V_{stat} at ~3850 V), the voltage is fixed at 3500 V. (2) The oxygen is introduced in the chamber to ~$1.1x10^{-7}$ mbar. (3) After 100 min, the O_2 is pumped out of the chamber. (4) The automatic field evaporation is re-activated with a targeted detection rate of 2%. The V_{stat} increases up to ~3900 V. During the entire experiment, the pulsed voltage V_{pulse} is fixed at 1314 V. **B.** Detection rate and Pressure plot of the same experiment. After fixing the voltage and introducing oxygen gas in the chamber, the detection rate quickly increases up to 12% before decreasing and stabilizing between 5 and 6%. The gas introduction is manually controlled through a leak valve. A slight pressure correction in mid experiment of ~$1.0x10^{-8}$ mbar instantly decreases the detection rate.

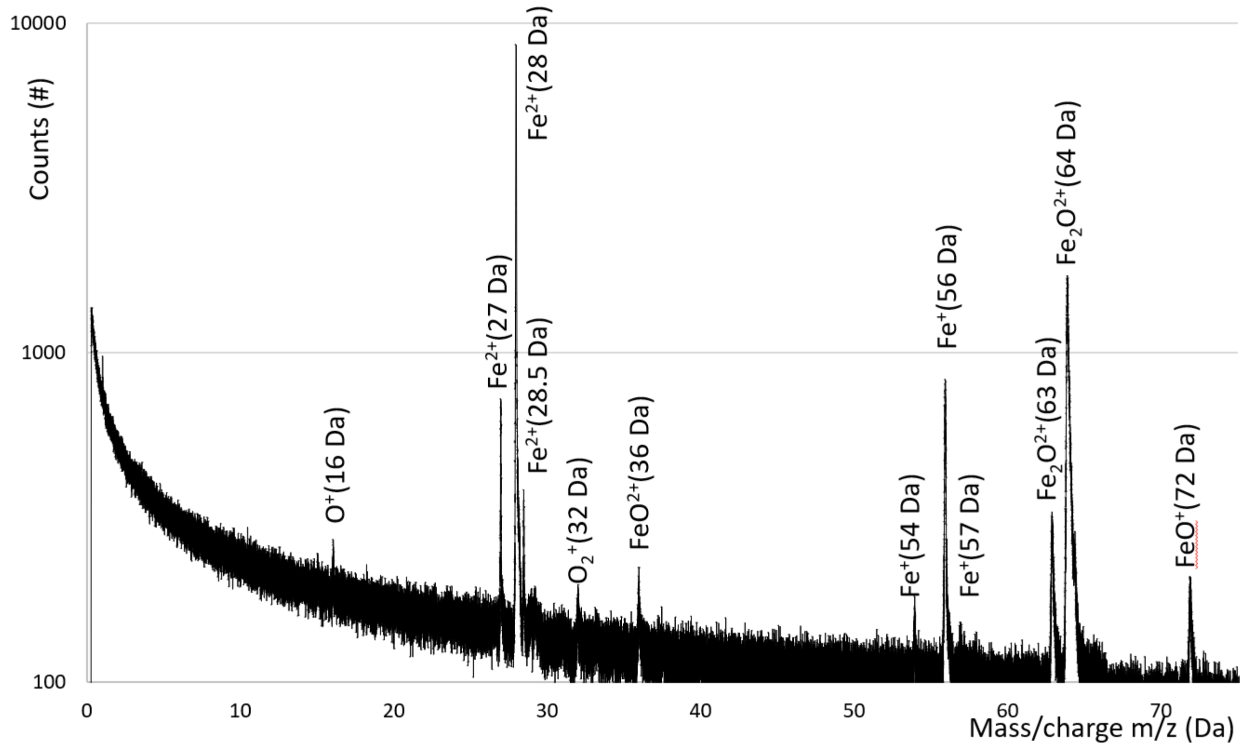

Figure S25: Mass spectra of the Environmental Atom Probe experiment.

Bibliography

[1] J.-S. McEwen, P. Gaspard, Y. De Decker, C. Barroo, T. Visart de Bocarmé, and N. Kruse, "Catalytic reduction of NO_2 with hydrogen on Pt field emitter tips : kinetic instabilities on the nanoscale" *Langmuir* **2010**, 26, 16381–16391.

[2] M.F.H. van Tol, A. Gielbert, R.M. Wolf, A.B.K. Lie, and B.E. Nieuwenhuys, "The striking difference in the behaviour of Rh and Pt towards their interaction with CO_2" *Surf. Sci.* **1993**, 287-288, 201–207.

[3] M.F.H. van Tol, A. Gielbert, and B.E. Nieuwenhuys, "The adsorption and dissociation of CO_2 on Rh" *Appl. Surf. Sci.* **1993**, 67, 166–178.

[4] G.K.L. Cranstoun and J.T. Lynch, "Field ion microscope studies of the oxidation of iron" *Appl. Surf. Sci.* **1980**, 5, 161–179.

[5] J.-S. McEwen, P. Gaspard, T. Visart De Bocarmé, and N. Kruse, "Oscillations and bistability in the catalytic formation of water on rhodium in high electric fields" *J. Phys. Chem. C* **2009**, 113, 17045–17058.

[6] S. Owczarek, S.V. Lambeets, R. Bryl, C. Barroo, O. Croquet, L. Markowski and T. Visart de Bocarmé, "Oxygen adsorption, subsurface oxygen layer formation and reaction with hydrogen on surfaces of a Pt–Rh alloy nanocrystal" *Top. Catal.* **2020**, 63, 1522–1531.

[7] S.V. Lambeets, C. Barroo, S. Owczarek, L. Jacobs, N. Gilis, N. Kruse, T. Visart de Bocarmé "Adsorption and hydrogenation of CO_2 on Rh nanosized crystals demonstration of the role of inter-facet oxygen spillover and comparative studies with O_2, N_2O and CO" *J. Phys. Chem. C* **2017**, 121, 16238–16429.

[8] J.-S. McEwen, P. Gaspard, F. Mittendorfer, T. Visart de Bocarmé, and N. Kruse, "Field-assisted oxidation of rhodium" *Chem. Phys. Lett.* **2008**, 452, 133–138.

[9] C. Barroo, Y. De Decker, L. Jacobs, and T. Visart de Bocarmé, "Nonlinear behavior during NO_2 hydrogenation on a nanosized Pt-Rh catalyst sample" *Appl. Surf. Sci.* **2017**, 412, 564–570.

[10] S.V. Lambeets, E.J. Kautz, M.G. Wirth, G.J. Orren, A. Devaraj, and D.E. Perea, "Nanoscale perspectives of metal degradation via in situ atom probe tomography" *Top. Catal.* **2020**, 63, 1606–1622.

[11] N.M.H. Janssen, M.F.H. van Tol, and B.E. Nieuwenhuys, "The interaction of oxygen with Rh studied by field electron microscopy" *Appl. Surf. Sci.* **1994**, 74, 1–12.

[12] V.V. Gorodetskii, V.I. Elokhin, J.W. Bakker, and B.E. Nieuwenhuys, "Field electron and field ion microscopy studies of chemical wave propagation in oscillatory reactions on platinum group metals" *Catal. Today* **2005**, 105, 183–205.

[13] M. Rezeq, J. Pitters, and R. Wolkow, "Tungsten nanotip fabrication by spatially controlled field-assisted reaction with nitrogen" *J. Chem. Phys.* **2006,** 124, 204716.

[14] B.E. Nieuwenhuys, "Correlation between work function change and degree of electron back-donation in the adsorption of carbon monoxide and nitrogen on group VIII metals" *Surf. Sci.* **1981**, 105, 505–516.

[15] V.V. Gorodetskii and B.E. Nieuwenhuys, "Chemisorption and dissociation of carbon monoxide on rhodium surfaces" *Surf. Sci.* **1981**, 105, 299–312.

[16] S. Katnagallu, F.F. Morgado, I. Mouton, B. Gault and L.T. Stephenson, "Three-dimensional atomically resolved analytical imaging with a field ion microscope" *Microsc. Microanal.* **2022**, 28, 1264–1279.

[17] B. Klaes, J. Renaux, R. Lardé, F. Delaroche, F. F. Morgado, L.T. Stephenson, B. Gault and F. Vurpillot, "Analytical three-dimensional field ion microscopy of an amorphous glass FeBSi" *Microsc. Microanal.* **2022**, 28, 1280–1288.

[18] Y. Hong, A. Hensley, J.-S. McEwen, and Y. Wang, "Perspective on catalytic hydrodeoxygenation of biomass pyrolysis oils: essential roles of Fe-based catalysts" *Catal. Letters* **2016**, 146, 1621–1633.

[19] K.A. Rogers and Y. Zheng, "Selective deoxygenation of biomass-derived bio-oils within hydrogen-modest environments: a review and new insights" *ChemSusChem* **2016**, 9, 1750–1772.

[20] J. Bray, A. J. R. Hensley, G. Collinge, F. Che, Y. Wang, and J.-S. McEwen, "Modeling the adsorbate coverage distribution over a multi-faceted catalytic grain in the presence of an electric field: O/Fe from first principles" *Catal. Today* **2018**, 312, 92–104.

[21] X. Wen and J. Guan, "Recent advancement in the electrocatalytic synthesis of ammonia" *Nanoscale* **2020**, 12, 8065–8094.

[22] M. Wan, H. Yue, J. Notarangelo, H. Liu, and F. Che, "Deep learning-assisted investigation of electric field–dipole effects on catalytic ammonia synthesis" *JACS Au* **2022**, 2, 1338–1349.

[23] F. Che, J.T. Gray, S. Ha, N. Kruse, S.L. Scott, and J.-S. McEwen, "Elucidating the roles of electric fields in catalysis: a perspective" *ACS Catal.* **2018**, 8, 5153–5174.

[24] F. Che, J.T. Gray, S. Ha, and J.-S. McEwen, "Reducing reaction temperature, steam requirements, and coke formation during methane steam reforming using electric fields: a microkinetic modeling and experimental study" *ACS Catal.* **2017**, 7, 6957–6968.

[25] M.L. Karahka and H.J. Kreuzer, "New physics and chemistry in high electrostatic fields" *Surf. Sci.* **2016**, 643, 164–171.
[26] H.J. Kreuzer, "Field-induced chemistry in catalysis: high pressure and high fields" *Top. Catal.* **2020**, 63, 1497–1501.
[27] S.M. Kathmann, "Electric fields and potentials in condensed phases" *Phys. Chem. Chem. Phys.* **2021**, 23, 23836–23849.
[28] J.R. Oppenheimer, "Three notes on the quantum theory of aperiodic effects" *Phys. Rev.* **1928**, 13, 66–81.
[29] T.F. Kelly, T.T. Gribb, J.D. Olson, R.L. Martens, J.D. Shepard, S.A. Wiener, T.C. Kunicki, R.M. Ulfig, D.R. Lenz, E.M. Strennen, E. Oltman, J.H. Bunton and D.R. Strait, "First data from a commercial local electrode atom probe (LEAP)" *Microsc. Microanal.* **2004**, 10, 373–383.
[30] R.M. Ulfig, D.J. Larson and S. Gerstl, "Advantages of using a digital detector for field ion microscopy" *Microsc. Microanal.* **2008**, 14, 124–125.
[31] M.K. Miller, "Implementation of the optical atom probe" *Surf. Sci.* **1992**, 266, 494–500.
[32] B. Deconihout, L. Renaud, G. Da Costa, M. Bouet, A. Bostel, and D. Blavette, "Implementation of an optical TAP: preliminary results" *Ultramicroscopy* **1998**, 73, 253–260.
[33] B. Gault, M.P. Moody, J.M. Cairney, and S.P. Ringer, *Atom probe microscopy*, Springer. New York Heidelberg Dordrecht London, **2012**.
[34] M.K. Miller, A. Cerezo, M.G. Hetherington, and G.D.W. Smith, *Atom probe field ion microscopy*, Claredon Press Oxford UK, **1996**.
[35] M. Wada, "On the thermally activated field evaporation of surface atoms" *Surf. Sci.* **1984**, 145, 451–465.
[36] T.T. Tsong, "Field ion image formation" *Surf. Sci.* **1978**, 70, 211–233.
[37] T. Ohnuma, "Surface diffusion of Fe and Cu on Fe (001) under electric field using first-principles calculations" *Microsc. Microanal.* **2019**, 25, 547–553.
[38] T. Ohnuma, "First-principles calculation of the evaporation field and roll-up effect of M (M = Fe, Cu, Si, and Mn) on the Fe (001) and Fe step structure" *Microsc. Microanal.* **2022**, 28, 1181–1187.
[39] J. Qi, C. Oberdorfer, W. Windl, and E.A. Marquis, "Ab initio simulation of field evaporation" *Phys. Rev. Mater.* **2022**, 6, 093602.
[40] R.M. Scanlan, D. L. Styris, and D.N. Seidman, "An in situ field ion microscope study of irradiated tungsten" *Philos. Mag. A J. Theor. Exp. Appl. Phys.* **1971**, 23, 1439–1457.
[41] K. Chibane and R. G. Forbes, "The temperature dependence of evaporation field for Gomer-type field-evaporation mechanisms" *Surf. Sci.* **1982**, 122, 191–215.
[42] M. Gruber, F. Vurpillot, A. Bostel, and B. Deconihout, "Field evaporation: A kinetic Monte Carlo approach on the influence of temperature" *Surf. Sci.* **2025**, 605, 2025–2031, 2025.
[43] F.C. von der Lage and H.A. Bethe, "A method for obtaining electronic eigenfunctions and eigenvalues in solids with an application to sodium" *Phys. Rev.* **1947**, 71, 612–622.
[44] R. Gomer, *Field Emission and Field Ionization*, Harvard Un. American Institute of Physics, **1993**.
[45] M. Wada, N. Akaiwa, and T. Mori, "Field evaporation of iron in neon and in hydrogen and its rate-controlling processes" *Philos. Mag. A* **1987**, 55, 389–403.
[46] H. Yanagisawa, V. Zadin, K. Kunze, C. Hafner, A. Aabloo, D.E. Kim, M.F. Kling, F. Djurabekova, J. Osterwalder and W. Wuensch "Laser-induced asymmetric faceting and growth of a nano-protrusion on a tungsten tip" *APL Photonics* **2016**, 1, 091305.
[47] E. Müller and T.T. Tsong, *Field ion microscopy principles and applications*, 1st ed., Elsevier Publishing Company Inc., New York, **1969**.
[48] H.J. Kreuzer and R.L.C. Wang, "Resonant field ionization: a new imaging mechanism in the field ion microscope" *Zeitschrift für Phys. Chemie* **1997**, 202, 127–138.
[49] A.J.R. Hensley, I. deJoode, Y. Wang, and J.-S. McEwen, "Identifying trends in the field ionization of diatomic molecules over adsorbate covered Pd(331) surfaces" *Top. Catal.* **2020**, 63, 1510–1521.
[50] C. Nowak, G. Schmitz, and R. Kirchheim, "Electric field effect on low temperature nanoscale oxidation" *Surf. Sci.* **2010**, 604, 641–648.
[51] I.E. McCarroll, D. Haley, S. Thomas, M.S. Meier, P.A.J. Bagot, M.P. Moody, N. Birbilis and J.M. Cairney, "The effect of hydrogen on the early stages of oxidation of a magnesium alloy" *Corros. Sci.* **2020**, 165, 108391.
[52] G.K.L. Cranstoun and J. Anderson, "Hydrogen promoted corrosion of tungsten by oxygen in an electric field: a field ion microscope study" *Surf. Sci.* **1973**, 35, 319–335.
[53] T.L. Martin, C. Coe, P.A.J. Bagot, P. Morrall, G.D.W. Smith, T. Scott and M.P. Moody, "Atomic-scale studies of uranium oxidation and corrosion by water vapour" *Sci. Rep.* **2016**, 6, 25618.

[54] B. Gault, A. Saksena, X. Sauvage, P. Bagot, L.S. Aota, J. Arlt, L.T. Belkacemi, T. Boll, Y.-S. Chen, L. Daly, M.B. Djukic, J.O. Douglas, M.J. Duarte, P.J. Felfer, R.G. Forbes, J. Fu, H.M. Gardner, R. Gemma, S.S.A. Gerstl, Y. Gong, G. Hachet, S. Jakob, B.M. Jenkins, M.E. Jones, H. Khanchandani, P. Kontis, M. Kramer, M. Kuhbach, R.K.W. Marceau, D. Mayweg, K.L. Moore, V. Nallathambi, B.C. Ott, J.D. Poplawsky, T. Prosa, A. Pundt, M. Saha, T.M. Schwarz, Y. Shang, X. Shen, M. Vrellou, Y. Yu, Y. Zhao, H. Zhao and B. Zou, "Towards establishing best practice in the analysis of hydrogen and deuterium by atom probe tomography" *Microsc. Microanal*. **2024**, 30, 1205-1220.

[55] M. Karahka, Y. Xia, and H. J. Kreuzer, "The mystery of missing species in atom probe tomography of composite materials" *Appl. Phys. Lett*. **2015**, 107, 062105.

[56] E.W. Müller, S. Nakamura, O. Nishikawa, and S. B. McLane, "Gas-surface interactions and field-ion microscopy of nonrefractory metals" *J. Appl. Phys*. **1965**, 36, 2496–2503.

[57] D.R. Kingham, "The post-ionization of field evaporated ions: A theoretical explanation of multiple charge states" *Surf. Sci*. **1982**, 116, 273–301.

[58] L. Tegg, L. T Stephenson and J.M. Cairney, "Estimation of the electric field in atom probe tomography experiments using charge state ratios" *Microsc. Microanal*. **2024**, 30, 466–475.

[59] G. Kresse and J. Hafner, "Ab initio molecular dynamics for liquid metals" *Phys. Rev. B* **1993**, 47, 558–561.

[60] G. Kresse and J. Furthmüller, "Efficient iterative schemes for ab initio total-energy calculations using a plane-wave basis set" *Phys. Rev. B* **1996**, 54, 11169–11186.

[61] G. Kresse and D. Joubert, "From ultrasoft pseudopotentials to the projector augmented-wave method" *Phys. Rev. B* **1999**, 59, 1758–1775.

[62] P.E. Blöchl, "Projector augmented-wave method" *Phys. Rev. B* **1994***,* 50, 17953–17979.

[63] M. Methfessel and A.T. Paxton, "High-precision sampling for Brillouin-zone integration in metals" *Phys. Rev. B* **1989**, 40, 3616–3621.

[64] B. Hammer, L. B. Hansen, and J. K. Nørskov, "Improved adsorption energetics within density-functional theory using revised Perdew-Burke-Ernzerhof functionals" *Phys. Rev. B* **1999**, 59, 7413–7421.

[65] A.J.R. Hensley, K. Ghale, C. Rieg, T. Dang, E. Anderst, F. Studt, C.T. Campbell, J.-S. McEwen and Y. Xu, "DFT-based method for more accurate adsorption energies: an adaptive sum of energies from RPBE and vdW density functionals" *J. Phys. Chem. C* **2017**, 121, 4937–4945.

[66] H.J. Monkhorst and J.D. Pack, "Special points for Brillouin-zone integrations" *Phys. Rev. B* **1976**, 13, 5188–5192.

[67] W.P. Davey, "Precision measurements of the lattice constants of twelve common metals" *Phys. Rev*. **1925**, 25, 753–761.

[68] T. Ossowski and A. Kiejna, "Structure and energetics of FeO/Fe(001) interfaces" *J. Phys. Condens. Matter* **2023**, 35, 465001.

[69] J. Neugebauer and M. Scheffler, "Theory of adsorption and desorption in high electric fields" *Surf. Sci*. **1993**, 287–288, 572–576.

[70] P.J. Feibelman, "Surface-diffusion mechanism versus electric field: Pt/Pt(001)" *Phys. Rev. B* **2001**, 64, 125403.

[71] K. Sillar, A. Kundu, and J. Sauer, "Ab initio adsorption isotherms for molecules with lateral interactions: CO_2 in metal-organic frameworks" *J. Phys. Chem. C* **2017**, 121, 12789–12799.

[72] P.M. Chaikin and T.C. Lubensky, *Principles of Condensed Matter Physics*. Cambridge: Cambridge University Press, **1995**.

[73] L. Xu, D. Kirvassilis, Y. Bai, and M. Mavrikakis, "Surface science atomic and molecular adsorption on Fe (110)" *Surf. Sci*. **2018**, 667, 54–65.

[74] P. Błoński, A. Kiejna, and J. Hafner, "Theoretical study of oxygen adsorption at the Fe(110) and (100) surfaces" *Surf. Sci*. **2005**, 590, 88–100.

[75] H.J. Kreuzer and S.H. Payne, "*Theoretical approaches to the kinetics of adsorption, desorption, and reactions at surfaces*" in *Computational Methods in Surface and Colloid Science*, 1st ed., CRC Press, **2000**, pp. 439–479.

[76] J.-S. McEwen, P. Gaspard, T. Visart de Bocarmé, and N. Kruse, "Electric field induced oscillations in the catalytic water production on rhodium: A theoretical analysis" *Surf. Sci*. **2010**, 604, 1353–1368.

[77] Y. Sakisaka, T. Miyano, and M. Onchi, "Electron-energy-loss-spectroscopy study of oxygen chemisorption and initial oxidation of Fe(100)" *Phys. Rev. B* **1984**, 30, 6849–6855.
